# Coordinated Reset Vibrotactile Stimulation Induces Sustained Cumulative Benefits in Parkinson’s Disease

**DOI:** 10.3389/fphys.2021.624317

**Published:** 2021-04-06

**Authors:** Kristina J. Pfeifer, Justus A. Kromer, Alexander J. Cook, Traci Hornbeck, Erika A. Lim, Bruce J. P. Mortimer, Adam S. Fogarty, Summer S. Han, Rohit Dhall, Casey H. Halpern, Peter A. Tass

**Affiliations:** ^1^Department of Neurosurgery, Stanford University School of Medicine, Stanford, CA, United States; ^2^Engineering Acoustics, Inc., Casselberry, FL, United States; ^3^Department of Neurology, Stanford University School of Medicine, Stanford, CA, United States; ^4^Quantitative Sciences Unit, Stanford University School of Medicine, Stanford, CA, United States; ^5^Center for Neurodegenerative Disorders, Department of Neurology, University of Arkansas for Medical Sciences, Little Rock, AR, United States

**Keywords:** coordinated reset, vibrotactile stimulation, Parkinson’s disease, desynchronization, cumulative effects, beta band power, sensorimotor

## Abstract

**Background:**

Abnormal synchronization of neuronal activity in dopaminergic circuits is related to motor impairment in Parkinson’s disease (PD). Vibrotactile coordinated reset (vCR) fingertip stimulation aims to counteract excessive synchronization and induce sustained unlearning of pathologic synaptic connectivity and neuronal synchrony. Here, we report two clinical feasibility studies that examine the effect of regular and noisy vCR stimulation on PD motor symptoms. Additionally, in one clinical study (study 1), we examine cortical beta band power changes in the sensorimotor cortex. Lastly, we compare these clinical results in relation to our computational findings.

**Methods:**

*Study 1* examines six PD patients receiving noisy vCR stimulation and their cortical beta power changes after 3 months of daily therapy. Motor evaluations and at-rest electroencephalographic (EEG) recordings were assessed off medication pre- and post-noisy vCR. *Study 2* follows three patients for 6+ months, two of whom received daily regular vCR and one patient from study 1 who received daily noisy vCR. Motor evaluations were taken at baseline, and follow-up visits were done approximately every 3 months. *Computationally*, in a network of leaky integrate-and-fire (LIF) neurons with spike timing-dependent plasticity, we study the differences between regular and noisy vCR by using a stimulus model that reproduces experimentally observed central neuronal phase locking.

**Results:**

*Clinically*, in both studies, we observed significantly improved motor ability. EEG recordings observed from study 1 indicated a significant decrease in off-medication cortical sensorimotor high beta power (21—30 Hz) at rest after 3 months of daily noisy vCR therapy. *Computationally*, vCR and noisy vCR cause comparable parameter-robust long-lasting synaptic decoupling and neuronal desynchronization.

**Conclusion:**

In these feasibility studies of eight PD patients, regular vCR and noisy vCR were well tolerated, produced no side effects, and delivered sustained cumulative improvement of motor performance, which is congruent with our computational findings. In study 1, reduction of high beta band power over the sensorimotor cortex may suggest noisy vCR is effectively modulating the beta band at the cortical level, which may play a role in improved motor ability. These encouraging therapeutic results enable us to properly plan a proof-of-concept study.

## Introduction

While the hallmark of idiopathic Parkinson’s disease (PD) is motor impairment, research suggests the underlying mechanism for this impairment is caused by abnormal neuronal synchrony within dopaminergic brain circuits (Hammond et al., 2007). Coordinated reset (CR) stimulation aims at long-lasting desynchronization by remodeling synaptic connectivity using specific spatiotemporal stimulus patterns (Tass and Majtanik, 2006). Computationally, it was shown that CR-induced desynchronization may reduce plastic synaptic weights, in this way causing long-lasting desynchronization and ultimately moving neural networks from stable synchronized and strong synaptically coupled states to stable desynchronized and weakly coupled states (Tass and Majtanik, 2006; Kromer and Tass, 2020). To this end, CR employs spike timing-dependent plasticity (STDP), a basic learning mechanism that modifies the strength of synapses according to the relative timing of their corresponding neurons’ presynaptic and postsynaptic spikes or bursts (Markram et al., 1997; Bi and Poo, 1998; Abbott and Nelson, 2000; Caporale and Dan, 2008). CR deep brain stimulation (CR-DBS) delivered to the subthalamic nucleus (STN) for 2 h per day during 5 consecutive days induced acute as well as sustained motor improvement lasting for several weeks in parkinsonian monkeys (Tass et al., 2012; Wang et al., 2016). CR-DBS administered to the STN in six PD patients for 4 h per day for 3 days caused a significant and cumulative reduction of STN beta band activity and a correlated significant improvement of motor function (Adamchic et al., 2014).

In this study, we administer a novel noninvasive CR technique called vibrotactile CR (vCR) fingertip stimulation (Tass, 2017) and explore its effects on motor ability and cortical activity in PD patients. Synchronization of cortical activity is quantified by means of electroencephalographic (EEG) power (Ebersole and Milton, 2003). Following the somatosensory pathway, vibrotactile stimulation results in enhanced neuronal activity of somatic sensory ventral caudal (Vc) thalamic relay neurons (Weiss et al., 2009), as well as in the primary somatosensory cortex (SI), as shown in macaque monkeys (Harvey et al., 2013). Furthermore, neuronal activity is phase locked to skin indentation oscillations (Weiss et al., 2009; Harvey et al., 2013), which allows one to modify collective neuronal discharge patterns by controlling the timing of discharges of stimulated subpopulations (Tass, 1999). To which extent this activity propagates to other cortical areas or the basal ganglia region is still not completely understood. Stimulation-related activity in the SI may propagate to motor areas *via* corticocortical connections, which has been observed in mammals across species (Jones and Powell, 1968; Jones and Powell, 1969; Mao et al., 2011). Furthermore, stimulation of the SI has been shown to elicit responses of motor cortical neurons (Kaneko et al., 1994). It is also well documented that the STN receives excitatory input from the cortical sensorimotor region *via* the hyperdirect pathway (Hartmann-von Monakow et al., 1978; Nambu et al., 1996), as well as from the thalamic intralaminar subnuclei CM/Pf (Sugimoto et al., 1983; Kita et al., 2016). It has been shown that neuronal activity may propagate among thalamic nuclei (Crabtree and Isaac, 2002; Crabtree, 2018). Furthermore, vibratory input may feed into motor basal ganglia thalamocortical circuits through different projections, e.g., through thalamo(Vc)-cortico(SI)-striatal projections. For instance, in squirrel monkeys, it was shown by electrophysiological recordings as well as tracer injection and histology that the SI feeds into the basal ganglia by projecting to the striatum (Flaherty and Graybiel, 1991).

Of particular interest is the impact of vibratory stimulation on cortical rhythms. For instance, muscle vibration can cause a reduction in motor threshold, as measured by motor-evoked potentials derived from transcranial magnetic stimulation (Rosenkranz and Rothwell, 2003). Additionally, when participants are at rest, vibration applied to the wrist induces cortical alpha and beta power suppression over the sensorimotor cortex (Seo et al., 2019). Reduced cortical beta power over the sensorimotor cortex during motor preparation, execution, and motor imagery suggests activation of this area (Neuper et al., 1999; Neuper and Pfurtscheller, 2001; Neuper et al., 2006). These findings indicate that vibratory stimulation alone can cause activation of the sensorimotor cortex through activation of afferent pathways *via* vibratory stimulation.

Other vibratory stimulation techniques include whole-body vibration (WBV). WBV has been found to improve performance during upper body exercise (Marín et al., 2013) and to reduce motor symptoms in PD patients (Haas et al., 2006; Ebersbach et al., 2008; King et al., 2009). However, a recent meta-analysis suggests that the symptom-reducing effects are inconsistent (Dincher et al., 2019) and do not improve certain components of gait or balance (Lau et al., 2011). Studies examining the cortical activations involved, especially within the sensorimotor cortex, during and after vibration therapy in PD have not been thoroughly investigated. It may be that WBV does not have clear therapeutic benefits because it lacks effective application parameters that target specific pathological brain regions, while CR effectively reduces PD symptoms both behaviorally (Tass et al., 2012; Syrkin−Nikolau et al., 2018) and on the neuronal level by targeting specific subpopulations (Adamchic et al., 2014).

In a first-in-human study, five idiopathic PD patients received vCR fingertip stimulation for 4 h per day on 3 consecutive days (Syrkin−Nikolau et al., 2018). Kinematic assessments revealed improved gait and bradykinesia during stimulation and after 1 month poststimulation. However, blinded video Unified Parkinson’s Disease Rating Scale (UPDRS) III scores (excluding items for rigidity and speech due to video constraints) did not show a significant change. As known from CR-DBS, a third of the pulse amplitude used for conventional high-frequency DBS caused significantly greater therapeutic effects (Tass et al., 2012), as CR requires separate stimulation of neuronal subpopulations (Tass, 2003; Tass and Majtanik, 2006). Accordingly, in the present studies, we used smaller vibration peak amplitudes (Adamchic et al., 2014) (0.06–0.10 mm) rather than the higher vibrational amplitude used in the first-in-human vCR study (0.35 mm; Syrkin−Nikolau et al., 2018).

Furthermore, we consider a randomized noisy vCR pattern. This is motivated by a previous computational study, wherein a network of leaky integrate-and-fire (LIF) neurons with STDP and electrical model stimuli, random reset (RR) stimulation was studied (Kromer and Tass, 2020). RR stimulation, achieved by adding spatial and temporal noise to the delivery mechanism of CR stimulation, may increase the robustness of long-term desynchronizing effects with respect to detuning the mean inter-stimulus interval relative to the dominant frequency of the abnormally synchronized neuronal target population (Kromer and Tass, 2020). We hypothesized that the robustness of vCR-induced long-lasting desynchronization might be improved by adding noise to stimulus delivery times.

In the present paper, we study the feasibility of vCR stimulation for the treatment of PD patients and report therapeutic effects required to design a rigorous proof-of-concept study. We present results of two clinical feasibility studies exploring the effects of regular (i.e., non-noisy) and noisy vCR on PD motor symptoms. We apply vCR to a total of eight PD patients. Study 1 examines six PD patients using a noisy vCR stimulation pattern and their cortical beta band activity changes over 3 months of daily vCR use. Study 2 follows three patients for 6+ months, two of whom received regular vCR and one patient from study 1 who received noisy vCR. Our aim in both studies was to understand how vCR affects PD patients by examining the cumulative effects of vCR, side effects, tolerability, and neuronal changes.

We compare our clinical results to computer simulations using the same neuronal network model as previously used for the design of RR stimulation (Kromer and Tass, 2020). However, we introduce a novel stimulus model for vibratory stimuli. This model is motivated by experimental data on the response of thalamic and cortical neurons to vibratory stimulation (Weiss et al., 2009; Harvey et al., 2013). In contrast to previous stimulus models (Popovych and Tass, 2012), vibratory stimuli do not result in a phase reset of the neuronal oscillations. To deliver noisy vCR stimulation, we added jitter to the stimulus delivery times within ranges that are favorable based on proprioceptive physiology and psychophysics.

## Materials and Methods

### Stimulation Patterns

**FIGURE 1:**
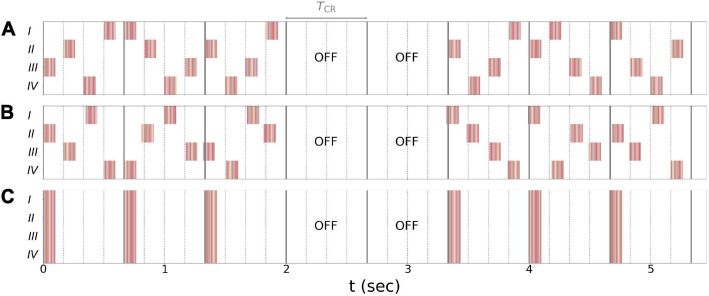
Stimulation patterns used throughout the paper. **(A)** Regular 3:2 ON-OFF coordinated reset with rapidly varying sequence (CR RVS) pattern. **(B)** Noisy 3:2 ON-OFF CR RVS pattern and 23.5% jitter. **(C)** Purely periodic multichannel stimulation. Gray lines indicate multiples of the vibrotactile coordinated reset (vCR) period *T*_CR_, and dotted lines indicate multiples of *T*_CR_/4 during individual CR periods. Roman numerals indicate fingertips on one hand. Stimulation bursts are marked red. Red vertical lines indicate maxima of *f*_vib_(*t*), Eq. (2), during individual bursts. Parameters: *f*_CR_ = 1.5 Hz ($\frac{T_{\text{CR}}}{4}$ = 166.7 ms), burst duration *100* ms, and *f*_burst_ = 250 Hz.

In the present paper, we explored vCR stimulation as treatment for PD. Three different stimulation patterns were considered: regular vCR (Tass, 2017), the novel noisy vCR, and purely periodic multichannel stimulation (PPMS) (Zeitler and Tass, 2018). Patients in study 1 were stimulated using the noisy vCR pattern and patients in study 2 using either the regular vCR or the noisy vCR pattern. In our computational study, we considered both regular and noisy vCR and compared the results to those seen with vibratory PPMS (vPPMS). The three stimulation patterns are illustrated in Figure 1 and were implemented as follows.

Regular vCR stimulation, Figure 1A, is characterized by the vCR period *T*_CR_, which sets the CR frequency *f*_CR_ = 1/*T*_CR_ at which individual fingertips received burst stimuli. Individual fingertips received stimuli at multiples of *T*_CR_/4 such that each fingertip received exactly one stimulus per CR period. Besides this constraint, stimuli were delivered to randomly selected fingertips. This type of CR stimulation is referred to as CR with rapidly varying sequences (CR RVS) in the literature (Zeitler and Tass, 2015). Additionally, we considered an *m:n* ON-OFF pattern by delivering stimuli for an ON-period of three CR periods, *T*_CR_, and paused the stimulation for an OFF period of two CR periods afterward (Lysyansky et al., 2011). A representative regular vibrotactile 3:2 ON-OFF CR RVS pattern, in the following denoted as regular vCR, is shown in Figure 1A.

Throughout this study, we also considered a noisy version of the vibrotactile 3:2 ON-OFF coordinated reset with rapidly varying sequence (CR RVS) pattern, described in the previous paragraph. The noisy vCR pattern was obtained by applying a random jitter to each stimulus onset time *s* (except for the very first stimulus). Each *s* was drawn from a uniform distribution $s\in \left[s_{0}-J\,\frac{T_{\text{CR}}}{8},s_{0}+J\,\frac{T_{\text{CR}}}{8}\right],$ where *s*_*0*_ is the original onset time, i.e., an integer multiple of *T*_CR_/4, and *J* is a jitter. *J* = 0% corresponds to regular vCR, while *J* > 0 leads to a noisy vCR pattern. In our clinical studies as well as in the computational model, we considered noisy vCR with *J* = 23.5%, a representative vibrotactile noisy 3:2 ON-OFF CR RVS pattern, in the following denoted as noisy vCR, is shown in Figure 1B.

Finally, we compared results for regular and noisy vCR stimulation to vPPMS as presented by Zeitler and Tass (2018). The latter is illustrated in Figure 1C. In contrast to vCR stimulation, vPPMS considers simultaneous stimulation of all fingertips and does not enforce phase shifts between the spiking activity of individual neuronal subpopulations (Tass, 2003; Zeitler and Tass, 2018). We therefore hypothesized poor performance with respect to long-lasting desynchronization effects.

### Vibrotactile Stimulation of Parkinson’s Disease Patients

For physiological design of vibrotactile glove and tactors, see Supplementary Vibrotactile Glove Material.

We used vibratory bursts of 100-ms length to achieve sufficient vibratory “loudness” (Green, 1976; Gescheider et al., 1999), while avoiding adaptation potentially caused by unnecessarily long vibratory bursts (Hahn, 1968; Goodwin, 2005; Leung et al., 2005). For noisy vCR, the jitter of stimulus times was constrained to avoid mutual masking of subsequent stimuli (Hollins et al., 1990).

For bilateral application of noisy vCR in PD patients, we used a mirrored delivery such that right and left fingers 2–5 were coincidently activated, respectively. This was done to avoid bilateral masking-like interference (Craig, 1985; Craig and Qian, 1997). In contrast, regular vCR was delivered to both hands in a non-mirrored manner, such that vibratory stimulus administration times were identical for both hands, but stimulus delivery was not coincident for fingers 2–5 of both hands. This mode was chosen to increase the spatial randomization, hypothesized to be more favorable to induce long-term synaptic decoupling (Kromer and Tass, 2020).

### Study 1: Impact of Noisy Vibrotactile Coordinated Reset on Motor Ability and Cortical Beta Power

#### Participants

Six patients were enrolled in the study after obtaining informed consent to the protocol approved by the institutional review board at Stanford University (CA, United States). All patients were clinically diagnosed with mild to moderate idiopathic PD by a staff movement disorders specialist (four men, two women, mean (*M*) age = 53.33 years, standard deviation (*SD*) = 10.78 years, *M* years since PD diagnosis = 9 years, *SD* = 4.3 years, Hoehn and Yahr stage 2–3, *M* = 2.33, *SD* = 0.51). Patients were further classified as tremor-dominant (*n* = 4), postural instability/gait difficulty (*n* = 1), or intermediate (*n* = 1) (Stebbins et al., 2013). All patients were cognitively assessed using the Scales for Outcomes in Parkinson’s Disease-COGnition (SCOPA-COG; Verbaan et al., 2011). No patient scored below the screening cutoff value ≤ 24 for PD dementia (*M* = 32.16, *SD* = 2.99). Exclusion criteria included atypical parkinsonism, presence of other neurological diseases, Hoehn and Yahr stage 1 or 5, previous brain surgery, history of skull fracture, or consumption of psychoactive medication that could alter EEG brain activity.

All patients took L-dopa or additional dopamine agonists. Prior to every visit, patients withdrew from PD medication using the following procedures: short-acting PD medication was withdrawn for 12 h, while long-lasting [Mirapex ER (extended release), Sinemet CR (controlled release), and Requip XL (extended release)] medication was withdrawn 24–48 h prior to the patient’s morning Movement Disorders Society-Unified Parkinson’s Disease Rating Scale III evaluations (MDS-UPDRS; Goetz et al., 2008a) and EEG recordings. Levodopa equivalent daily dose (LEDD; Tomlinson et al., 2010) was calculated for each patient prior to participation according to patient reports (*M* = 711.50, *SD* = 207.85). In addition, we collected daily medication diaries from patients online to observe medication intake. While we did not ask patients to reduce their daily medication, a 2-week average of LEDD prior to the 3-month follow-up visit was used to measure vCR effects on medication intake (*M* = 644.83, *SD* = 229.85; for patient demographics, see Supplementary Table 1).

#### Study Procedures

The following tests were administered on medication 1–2 weeks before vCR treatment (pretreatment screening) and 1–2 weeks before the 3-month post-vCR follow-up. These tests included the MDS-UPDRS (Goetz et al., 2008a) parts I (non-motor experiences of daily living), II (motor aspects of daily living), and IV (motor complications) and the Parkinson’s Disease Questionnaire-39 (PDQ-39; Jenkinson et al., 1997), which measures PD-specific quality of life over the past month.

**FIGURE 2:**
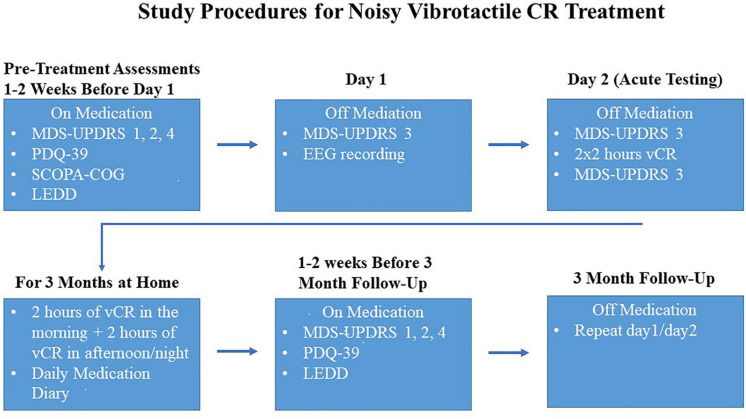
Study procedures were as follows: 1–2 weeks before the first visit, while on medication, Parkinson’s disease (PD) patients took the Movement Disorders Society-Unified Parkinson’s Disease Rating Scale (MDS-UPDRS) parts 1,2, and 4, Parkinson’s Disease Questionnaire-39 (PDQ-39), Scales for Outcomes in Parkinson’s Disease-COGnition (SCOPA-COG) and reported levodopa equivalent daily dose (LEDD). On the first visit, PD patients were off medication and the MDS-UPDRS 3 was administered, followed by an at-rest electroencephalography (EEG). Patients withdrew their medication again overnight and on day 2 patients were assessed with a morning MDS-UPDRS part 3, followed by 2 × 2 h of vibrotactile coordinated reset (vCR) stimulation and then an afternoon MDS-UPDRS part 3 (directly after 4 h of stimulation) to assess acute effects of vCR. After day 2, patients were instructed at home to do 2 h of vCR treatment in the morning and 2 h in the afternoon or night. The assessments described above were then repeated at 3 months of vCR treatment.

After the pre-assessment evaluations, PD patients were examined off medication over the course of 2 days using the following methods. Day 1 (baseline) consisted of a morning MDS-UPDRS III evaluation and an EEG recording (please note, on day 1 of the first visit, PD patients did NOT receive a full daily vCR session but did receive a short 10-min vCR stimulation period during the EEG recording). Day 2 consisted of a morning MDS-UPDRS III evaluation, followed by a vCR 2 × 2-h stimulation session with a 30–60-min break in between sessions. Immediately following 4 h of vCR stimulation, the afternoon MDS-UPDRS III evaluation occurred. Vibrotactile stimulation was delivered on digits 2–5 to both hands (excluding the thumbs). All MDS-UPDRS III evaluations were done under the supervision of a movement disorders specialist. This set of procedures was repeated at the 3-month follow-up; however, patients continued their 2 × 2-h vCR session after the EEG on the first day of the 3-month follow-up. After the first initial baseline visit, patients were sent home with the custom vibrotactile device and instructed to stimulate 2 h in the morning and 2 h in the afternoon or evening on both hands until the 3-month follow-up. For a detailed schematic of the study procedures, see Figure 2.

#### Electroencephalographic Acquisition and Recording Procedures

High-density EEG data were collected using an EGI Net Amps 400 amplifier and a 256-electrode Hydrocel Geodesic sensor net (Magstim, Electrical Geodesics, Inc., Eugene, OR, United States). Online EEG data were digitized at 1,000 samples per second and referenced to Cz. To ensure proper signal-to-noise ratio, impedances were kept below 50 kΩ. EEG recordings were done in a dimly lit soundproof Faraday chamber while participants sat comfortably in a reclining chair. Participants were recorded for a total of 30 min, which consisted of a 10-min at-rest pre-vCR baseline, a 10-min during vCR stimulation, followed by a 10-min post-vCR recording. Patients were instructed to alternate between 1-min eyes closed and 1-min eyes open in a counterbalanced fashion throughout each of the 10-min recordings.

#### Electroencephalographic Preprocessing

All EEG cleaning procedures were performed using Matlab R2019a and EEGlab v2019.1 (Delorme and Makeig, 2004). To minimize artifacts, only the eyes closed portions of data were used for analysis. The 1-min eyes closed epochs per condition were then chosen using the following principles. First, CR is most effective as a cumulative treatment over time (Hauptmann and Tass, 2009; Adamchic et al., 2014). Therefore, the last eyes closed epoch during vCR was used for analysis. In addition, the last 1-min eyes closed epoch pre-vCR was used as a comparison. Lastly, to measure the most immediate post-vCR EEG effects, the first eyes closed epoch post-vCR was used for analysis.

**FIGURE 3:**
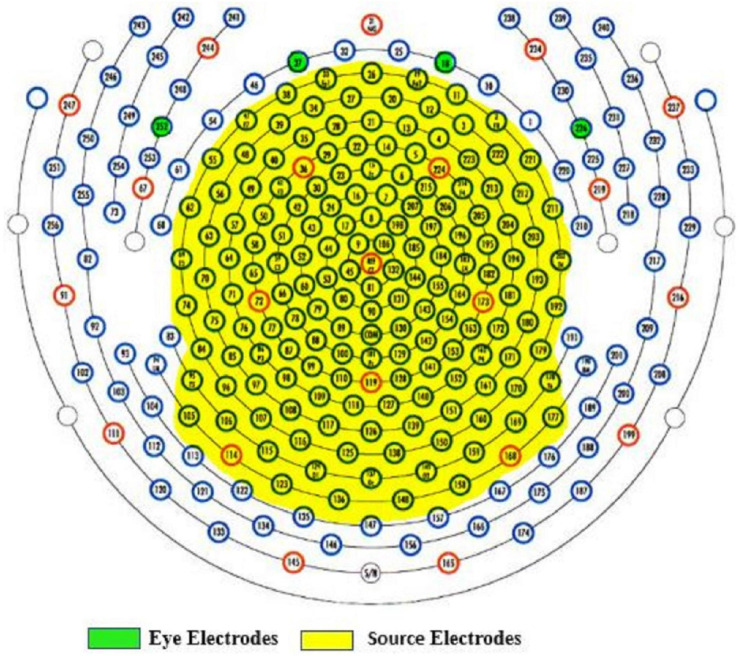
Yellow highlighted areas indicate selected electrodes for source estimation. Data from electrodes near or on the cheeks or close to the nape of the neck were removed to reduce noise. Vertical (E18, E37) and lateral (E252, E226) eye electrodes are highlighted in green.

The following procedures were administered to remove EEG artifacts. Data from electrodes near/on the cheeks or close to the nape of the neck were removed to reduce noise interference since these electrodes are more vulnerable to artifacts. Vertical and lateral eye electrodes were recorded from predefined areas according to the 256-electrode EGI sensor net (see Figure 3 for selected electrodes). A total of 171 electrodes including eye electrodes were used for subsequent analysis. EEG data were then average referenced and finite impulse response (FIR) filtered between 1 and 100 Hz, 6-dB octave with a 60-Hz notch. EEG artifacts were identified and removed using the clean_rawdata EEGlab plug-in (Mullen et al., 2015). This plug-in easily identifies and separates low-frequency drifts, flatlining, and artifact-ridden channels. Parameters for electrode exclusion were set to remove a channel if flatlining for more than 5 s, if high-frequency noise was beyond *SD* = 4 of the entire channel file, and if correlations with nearby electrodes fell below 0.7. Data portions whose variance was *SD* > 7 and/or 25% of electrodes went out of bounds relative to the overall data were automatically removed. In addition, artifact-ridden data identified from the clean_rawdata plug-in were visually inspected to confirm accuracy. For each EEG recording, no more than five electrodes were removed. The average length of data per 1-min epoch was: pre-vCR = 55.42 s, *SD* = 5.11 s, during-vCR = 52.75 s, *SD* = 7.73 s, post-vCR = 52.54 s, *SD* = 5.82 s. After electrode and artifact rejection, data were then re-average referenced and fastICA (Hyvarinen, 1999) was run to correct for eye blinks and excessive muscle activity. In all data sets, no more than three independent component analysis (ICA) components were removed. Bad electrodes were then added back into the data set using spherical spline interpolation (Perrin et al., 1989).

#### Source Signal Extraction

Cleaned EEG data, with eye channels removed for source estimation, were then imported into Brainstorm (Tadel et al., 2011). The following steps were taken to create and extract source signals. The default magnetic resonance imaging (MRI) anatomy ICBM152 model was used for all subjects. Surface envelopes for the scalp, inner skull, and outer skull using the boundary element method (BEM) were generated (1,922 vertices per layer). MRI tissue segmentation was generated using the computational anatomy toolbox (CAT) (Gaser et al., unpublished). Here, 15,000 vertices were generated on the cortex surface, and spherical registration was used. Since our baseline EEG data were meaningful, no noise modeling was used for our noise covariance matrix. Forward modeling was then computed using OpenMEEG implemented in Brainstorm, which is based on the symmetric BEM. EEG source estimation was computed using minimum norm imaging (MNI). Standardized low-resolution brain electromagnetic tomography analysis (sLORETA; Pascual-Marqui, 2002) was used as a method to measure cortical activity, and dipole orientations were constrained to the cortex.

Power spectral density (PSD) was calculated from source activity for each frequency band of interest (Delta: 2–4 Hz; Theta: 5–7 Hz; Alpha: 8–12 Hz; Low Beta: 13–16 Hz; Mid Beta: 17–20 Hz; High Beta: 21–30 Hz; and Gamma: 31–50 Hz) using the Welch method with a 2-s window overlapping by 50%. Relative power (RP) was calculated by taking the sum of each frequency band and dividing it by the total power across the spectrum (2–50 Hz).

**FIGURE 4:**
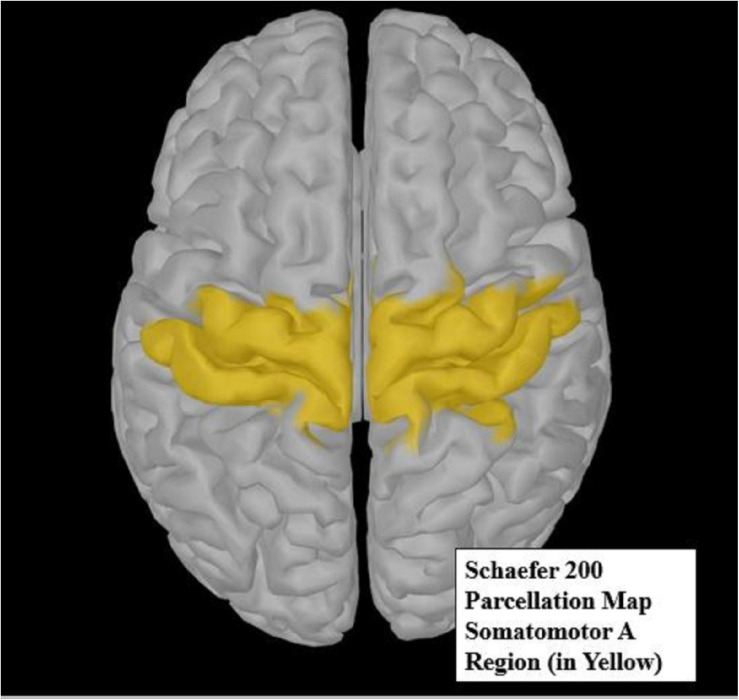
The yellow highlighted region of the Schaefer 200 parcellation map (Schaefer et al., 2018) represents the somatomotor A region of interest (ROI). This ROI was used for source analysis.

The Schaefer 200 parcellation map (Schaefer et al., 2018) was used to select a region of interest (ROI). The Schaefer parcellation map uses a gradient weighted Markov Random Field model that effectively produces homogeneous parcellations within cortical regions identified by histology or visuotopic functional MRI. The somatomotor A region (Figure 4) is broken up into 19 parcellations, all of which are considered distinct from other cortical boundaries. The somatomotor A region, which includes the hands and several other body parts but excludes auditory areas, was chosen for analysis, as it contains the most relevant regions for vibrotactile stimulation. Due to small sample size, we restricted our analysis only to the somatomotor A region rather than the whole cortex. The somatomotor A region can also be referred to the sensorimotor region, as it comprises both the primary motor and the primary somatosensory cortex. This region was chosen as the best possible area to extract sensorimotor activity, as vibration (Seo et al., 2019) and movement (Neuper et al., 1999) are known to activate the sensorimotor area.

#### Behavioral Data Extraction and Statistical Analysis

The MDS-UPDRS III was used as the main outcome variable for analysis. Subgroups of PD motor symptoms were subdivided based on a previous publication’s classifications (Li et al., 2018). The four subgroups of the MDS-UPDRS III included for analysis were: tremor (total of items 15–18), rigidity (item 3), bradykinesia (total of items 2, 4–9, and 14), and axial (total of items 1 and 9–13). Acute effects of vCR were defined as the comparison between morning MDS-UPDRS III scores (before daily stimulation) and afternoon MDS-UPDRS III (immediately after 4 h of stimulation) done on day 2 of the first visit and day 2 after 3 months of vCR treatment. The morning MDS-UPDRS III scores measured on day 1 of the first visit and day 1 at the 3-month follow-up visit were used to measure cumulative effects. Day 1 MDS-UPDRS III scores were chosen to analyze cumulative effects rather than day 2 scores, as patients were off medication for a prolonged amount of time on day 2. We did not want possible side effects of prolonged medication withdrawal to negatively affect our main cumulative results.

To compare mean differences between two dependent groups, a paired-samples t-test was used to analyze acute and cumulative effects. To check for clinical significance, we further compared both acute and cumulative results to minimal clinically important differences (MCID) on the MDS-UPDRS III (Horváth et al., 2015). Specifically, for acute effects, MCID scores were calculated by subtracting pretreatment (baseline) vCR MDS-UPDRS III scores from posttreatment vCR MDS-UPDRS III scores on the first visit of the second day. Cumulative MCID effects were measured by subtracting pretreatment (baseline) vCR MDS-UPDRS III scores from posttreatment vCR MDS-UPDRS III scores after 3 months of vCR treatment. These difference scores will be denoted as Delta MDS-UPDRS III = post-vCR MDS-UPDRS III minus pre-vCR MDS-UPDRS III. In addition, the MDS-UPDRS I, II, III, and IV, PDQ-39, and LEDD done at the pre-assessment visit and 1–2 weeks before the 3-month follow-up were analyzed using a paired-samples *t*-test. Lastly, we calculated percentage decreases in LEDD, which compared baseline LEDD and 3-month LEDD. Specifically, baseline LEDD was based on patient reports at the 1–2-week pre-study assessment, and the 3-month LEDD was based on a 2-week LEDD average collected from patient medication diaries prior to the 3-month visit.

#### Electroencephalographic Data Extraction and Statistical Analysis

Source signals were extracted by obtaining the mean relative frequency band power of all voxels within each parcellation of the somatomotor A region. All parcellations were then averaged to obtain a single quantity of relative power at each frequency band.

Acute effects for EEG data were defined as the comparison of each 1-min eyes closed epochs (as described above), which include pre-vCR, during-vCR, and post-vCR recordings done on day 1 of the first visit and repeated on day 1 after 3 months of vCR treatment. To compare within-subject differences in relative power mean scores between pre-, during, and post-recordings of acute sensorimotor source-converted EEG data done at baseline and 3 months, separate repeated-measures analyses of variance (RMANOVAs) were run on each frequency band.

Cumulative effects were defined as the comparison of the 1-min eyes closed pre-vCR baseline recording performed at the first study visit and the 1-min eyes closed baseline pre-vCR recordings done after 3 months of therapy. To compare relative power mean differences between two dependent groups, a paired-samples *t*-test was used to evaluate the two baseline sensorimotor source-converted EEG recordings.

### Study 2: Impact of Prolonged Vibrotactile Coordinated Reset Therapy on Parkinson’s Disease Motor Symptoms

To understand long-term cumulative effects of vCR, we followed three patients for 6+ months. Two patients received regular vCR, while one patient from study 1 received noisy vCR. Initially, study 1 was intended to be a 13-month trial; however, coronavirus disease 2019 (COVID-19) prevented study 1 from going beyond 3 months. One patient from study 1 was able to receive MDS-UPDRS III scores remotely from his/her movement disorders neurologist. Therefore, we included this patient in study 2 and examined long-term cumulative vCR effects within all three patients.

#### Participants

Three patients were enrolled in the study after obtaining informed consent to the protocol approved by the institutional review board at Stanford University (CA, United States). All patients were diagnosed with idiopathic PD by a staff movement disorders specialist. For every MDS-UPDRS III rating, each patient was off PD medication for at least 12 h. After the first initial baseline MDS-UPDRS III evaluation (pre-vCR therapy), patients received daily at-home vCR therapy. Follow-up MDS-UPDRS III evaluations were done approximately every 3 months across 1–3 days.

##### Patient 1

Patient 1 was in his/her early 70s and was diagnosed with PD 4 years prior to participation in this study (LEDD pre-study = 450 mg/day). Patient 1 received regular vCR (0.1 mm) for 338 days for 2 × 2 h per day, during the first 216 days to the (more affected) right hand and bilaterally thereafter. Each visit, the patient’s motor ability was tested in the morning (before daily stimulation) and in the afternoon (directly after 4 h of stimulation) off medication.

##### Patient 2

Patient 2 was in his/her mid-50s and was diagnosed with PD 10 years prior to the start of this study. At the start of vCR, patient 2’s reported LEDD was 2,700 mg/day, and he/she also took 2–3 vapes of cannabidiol/tetrahydrocannabidiol (CBD/THC). Patient 2 received bilateral regular vCR (0.1 mm) for 185 days for 2 h per day. Due to the patient’s reported adverse effects of medication withdrawal, this patient had off-medication assessments on the morning of the first day of each visit only.

##### Patient 3

Patient 3 was in his/her early 60s and diagnosed with PD 12 years prior to participation in the study. At the start of vCR therapy, patient 3’s reported LEDD was 920 mg/day. Patient 3 received bilateral noisy vCR (0.06 mm) 2 × 2 h a day for 185 days followed by a preplanned 1-month no-stimulation pause (but continuing pharmacological therapy) to assess long-lasting effects of vCR. After the preplanned 1-month follow-up, the patient continued vCR therapy but reduced his/her daily dose to a minimum of 2 h of stimulation three times per week for 2 months. Each visit, the patient was tested in the morning (before daily stimulation) and in the afternoon (directly after 4 h of stimulation) off medication.

#### Motor Symptom Evaluations and Statistical Analysis

The MDS-UPDRS III was used as the main outcome variable to evaluate motor symptoms by a trained movement disorders specialist. In addition, four subscores of the MDS-UPDRS III were analyzed and included: tremor, rigidity, bradykinesia, and axial, with all items selected in the same manner as in study 1. All motor scores obtained throughout the study were done off medication (≥12 h). Since each patient had a different number of vCR treatment days and number of MDS-UPDRS III scores taken, separate Pearson’s r correlations for each patient were used to quantify the linear relationship between morning MDS-UPDRS III scores and days of vibrotactile use. In addition, to observe acute effects for patients 1 and 3, we report difference scores by subtracting pretreatment vCR morning scores (≥8 h without vCR) from posttreatment vCR motor scores (immediately after 4 h of vCR) done at baseline and at approximately every 3-month visit. For patient 3, we also report difference scores after the 1-month pause in stimulation by subtracting 6-month MDS-UPDRS III scores from 7-month MDS-UPDRS III scores. Lastly, we report baseline and posttreatment LEDD individually for all patients, and for patient 2, we report CBD/THC amount and off times.

### Computational Study

Brain regions possessing excessive neuronal synchrony during PD were modeled using a network of excitatory LIF neurons with STDP (Kromer and Tass, 2020; Kromer et al., 2020). Details on the model are given in the Supplementary Material. Parameters were chosen such that a stable synchronized and a stable desynchronized state coexisted (Kromer and Tass, 2020; Kromer et al., 2020). Depending on their initial connectivity, networks approached either the synchronized state (strong initial connectivity) or the desynchronized state (weak initial connectivity) (see Supplementary Material for more details). Throughout the present paper, we present results on vibrotactile stimulation of networks that were prepared in the synchronized state.

#### Vibrotactile Stimulation of the Neuronal Network Model

We incorporated vibrotactile stimulation in our network of excitatory LIF neurons. Neurons were separated into four neuronal subpopulations, each processing afferent vibrotactile input from a single fingertip. Each subpopulation consisted of 25% of the neurons, and subpopulations did not overlap. Neurons were subject to inhomogeneous Poisson input with firing rate

(1) $$f_{i\,n\,p\,u\,t}\,\left(t\right)=f_{b\,g}+f_{v\,i\,b}\,\left(t\right).$$

The homogeneous part, *f*_bg_, models stochastic background input from other brain regions. When a vibrotactile burst was delivered to a fingertip, related neurons were subject to additional excitatory inhomogeneous Poisson input with firing rate *f*_vib_(*t*), see Eq. (1), that was present for the duration of the vibrotactile stimulation burst (duration *T=100* ms). Note that transmission delays between vibratory stimulus delivery and neuronal firing rate modulation did not affect the results in the computational model as long as signals from the four fingertips experience the same delay time.

Experiments reported phase locking between skin indentation oscillations and neuronal activity of cortical neurons in primates (Harvey et al., 2013) and thalamic neurons in humans (Weiss et al., 2009). This was accounted for by a periodic modulation of the input firing rate

(2) $$f_{v\,i\,b}\,\left(t\right)=A\,\left(1+\cos\left(2\,\pi \,f_{b\,u\,r\,s\,t}\,t\right)\right)$$

during vibrotactile stimulation of the fingertip. *A* is the amplitude of firing rate oscillations, and *f*_burst_ is the frequency of vibratory oscillations against the skin. The latter were assumed to be sinusoidal, such that *f*_vib_(*t*) was maximal at the highest first derivative of skin indentation oscillations (Weiss et al., 2009).

In experiments, the mean input firing rate *A*, obtained by time averaging Eq. (2) over time intervals long compared to 1/*f*_burst_, was controlled by the amplitude of skin indentation oscillations (Harvey et al., 2013). We therefore considered it as a free parameter. We measured *A* in units of *A*_0_, which is the amplitude at which the total postsynaptic current was sufficient to drive the neurons’ membrane potentials from reset to spiking threshold (see Supplementary Material for more details).

#### Measure of Synchrony

We recorded neuronal spike trains, and the degree of synchronization was quantified using the Kuramoto order parameter (Kuramoto, 1984)

(3) $$\rho \,\left(t\right)=\left|\frac{1}{N}\,\sum_{k=1}^{N}e^{-I\,\psi_{k}\,\left(t\right)}\right|.$$

ψ_k_(*t*) is the phase associated with neuron *k*. ψ_k_(*t*) increased linearly between consecutive spikes by a total amount of 2π per interspike interval (Rosenblum et al., 2001). Here, ρ(*t*)≈1 and ρ(*t*)≈0 indicate presence and absence of in-phase synchronization, respectively.

## Results

### Study 1: The Impact of Noisy Vibrotactile Coordinated Reset on Clinical and Electroencephalographic Data

#### Acute Effects of Noisy Vibrotactile Coordinated Reset on the Movement Disorders Society-Unified Parkinson’s Disease Rating Scale III

**FIGURE 5:**
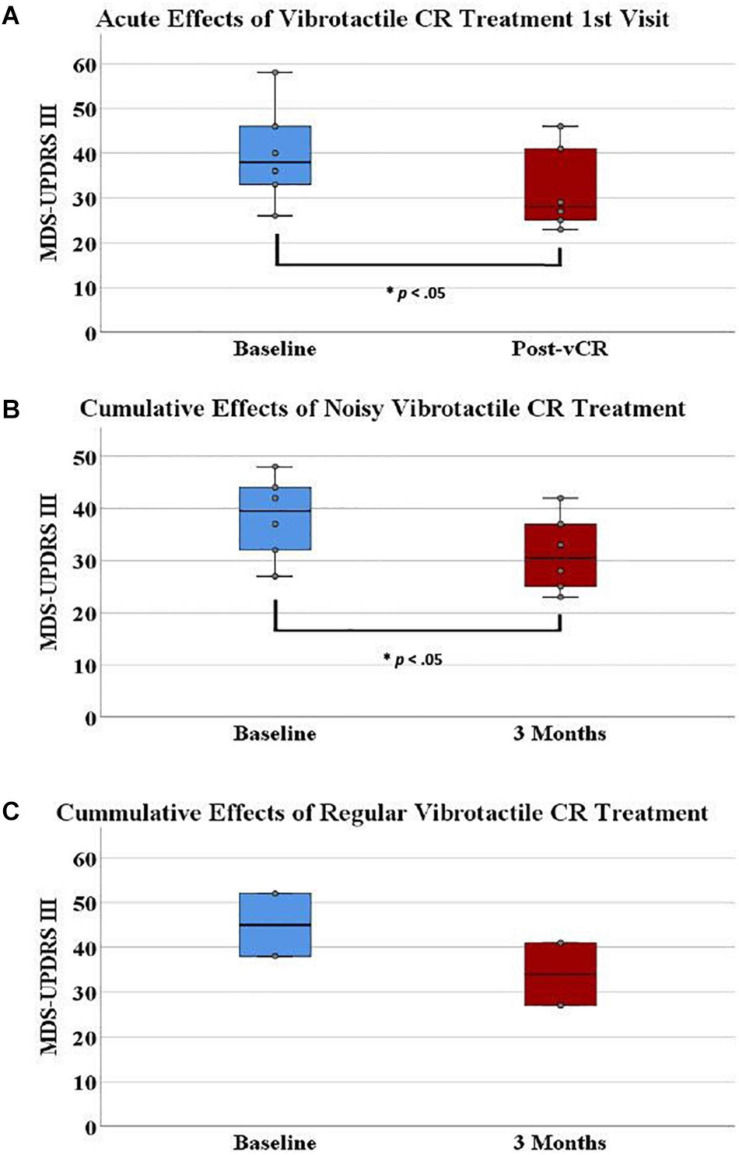
**(A)** On the first visit, Parkinson’s disease (PD) patients displayed a significant acute effect for vibrotactile coordinated reset (vCR) treatment. Specifically, Movement Disorders Society-Unified Parkinson’s Disease Rating Scale (MDS-UPDRS) III pretreatment scores (*M* = 39.833, SD = 11.14) significantly decreased after 4 h of stimulation (*M* = 31.833, SD = 9.38). **(B)** Additionally, MDS-UPDRS III pretreatment scores (*M* = 38.33 ± 7.86) significantly decreased after 3 months of vCR treatment (*M* = 32.33, *SD* = 7.80). Panel **(C)** represents baseline (*M* = 45, *SD* = 9.89) and 3-month (*M* = 34, *SD* = 9.89) MDS-UPDRS III data for the two patients in study 2 who received regular vCR. While no statistics can be used due to the small sample size, regular vCR results are represented visuallyfor comparison to the noisy vCR results. Regardless of vCR type, these findings suggest significant improvement of motor ability. Panels **(A,B)** show box plots, whereas the boxes in panel **(C)** simply comprise the two patients’ values.

In assessing the acute motor effects of noisy vCR at the outset of therapy, paired-samples *t*-tests revealed a significant effect for the MDS-UPDRS III [*N* = 6, *t*(5) = 4.297, *p* = 0.008, *SD* = 4.56]. Specifically, day 2 pretreatment scores (*M* = 39.833, *SD* = 11.14) decreased after 4 h of vCR stimulation (*M* = 31.833, *SD* = 9.38; Figure 5A). Axial symptom subscores showed a significant effect [*t*(5) = 4.719, *p* = 0.005, *SD* = 1.211], with day 2 pre-vCR scores (*M* = 5.33, *SD* = 2.42) decreasing after 4 h of stimulation (*M* = 3.0, *SD* = 2.68). Rigidity was also trending toward improvement [*t*(5) = 2.449, *p* = 0.058, *SD* = 2.0], with day 2 pretreatment scores (*M* = 10.166, *SD* = 4.16) decreasing after 4 h of stimulation (*M* = 8.166, *SD* = 2.9).

**FIGURE 6:**
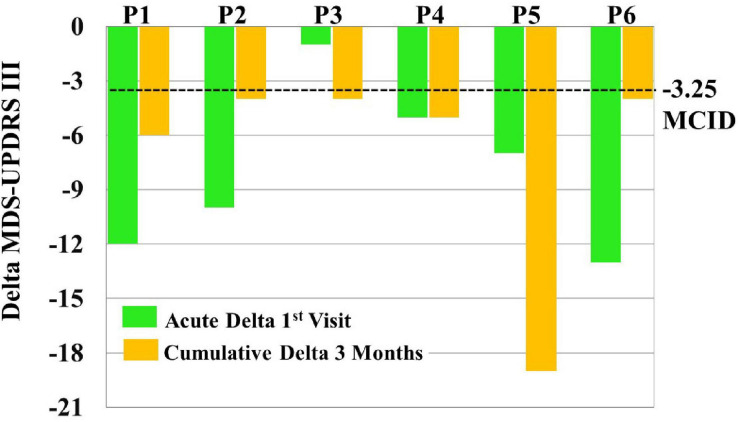
To assess clinical significance of acute and cumulative treatment outcomes, minimal clinically important differences (MCID = –3.25) were compared to Movement Disorders Society-Unified Parkinson’s Disease Rating Scale (MDS-UPDRS) score changes [i.e., Delta MDS-UPDRS III = post-vibrotactile coordinated reset (vCR) MDS-UPDRS III minus pre-vCR MDS-UPDRS III] obtained by subtracting pretreatment (baseline) vCR MDS-UPDRS III scores from posttreatment vCR MDS-UPDRS III scores on the first visit to measure acute effects (green bars) and by subtracting pretreatment (baseline) vCR MDS-UPDRS III scores from posttreatment vCR MDS-UPDRS III scores after 3 months of vCR treatment to measure cumulative effects (orange bars). For acute effects measured on the first visit, five out of six patients were able to clinically reduce MDS-UPDRS III after 4 h of vCR treatment. Additionally, all patients showed a clinically significant reduction of MDS-UPDRS III scores after 3 months of vCR treatment.

MCID for MDS-UPDRS III acute effects can be seen for each patient in Figure 6. Specifically, five out of six patients showed a clinically significant reduction of MDS-UPDRS III scores acutely on the first visit (i.e., a reduction in scores exceeding 3.25).

In assessing the acute MDS-UPDRS III effects of noisy vCR following the 3-month treatment period, a paired-samples t-test demonstrated a significant effect for rigidity subscores [*N* = 6, *t*(5) = 2.907, *p* = 0.034, *SD* = 0.983]. Specifically, day 2 pretreatment rigidity scores (*M* = 7.166, *SD* = 3.868) significantly decreased after 4 h of vCR stimulation (*M* = 6.00, *SD* = 3.098). No other significant differences were found for acute effects at 3 months.

#### Cumulative Effects of Noisy Vibrotactile Coordinated Reset on Clinical Data

In studying cumulative effects of noisy vCR following the 3-month treatment period, a paired-samples t-test revealed a significant effect for the overall MDS-UPDRS III [*N* = 6, *t*(5) = 2.890, *p* = 0.034, *SD* = 5.93]. Specifically, day 1 pretreatment scores (*M* = 38.33, *SD* = 7.86) significantly decreased after 3 months of vCR treatment (32.33, *SD* = 7.80; Figure 5B). Subscores for tremor showed a trending effect [*t*(5) = 2.314, *p* = 0.069, *SD* = 5.93], with day 1 pretreatment scores (*M* = 8.5, *SD* = 4.46) decreasing after 3 months of vCR treatment (*M* = 6.66, *SD* = 4.84). No other significant cumulative effects were found for the MDS-UPDRS I, II, and IV, PDQ-39, or LEDD pre- and post-3-month treatment. LEDD was reduced on average by 7.82% after 3 months of vCR treatment.

Minimal clinically important differences for MDS-UPDRS III cumulative effects can be seen for each patient in Figure 6. Specifically, all six patients experienced reductions in MDS-UPDRS III scores after 3 months of vCR treatment by a clinically significant amount (i.e., by more than 3.25 points; see Supplementary Video 1 for vCR effects in study 1).

#### Relative Power Source Results

One patient was unable to do the 3-month EEG recording due to COVID-19 restrictions, leaving a total of five study participants included for all analyses. However, for the acute analysis done on the first study visit, we ran two separate RMANOVAs, one of which included the participant who was unable to participate in the 3-month EEG recording (*N* = 6) and one analysis in which this patient was removed (*N* = 5). In both instances, no significant acute differences in relative power for any frequency band were found between pre-vCR, during-vCR, and post-vCR EEG recordings. Additionally, at the 3-month study visit, no significant acute differences in relative power were found in any frequency band between conditions.

**FIGURE 7:**
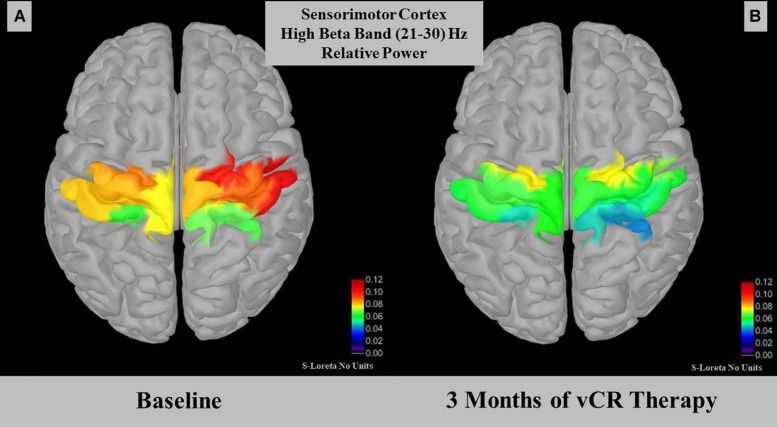
Displays relative power for the high beta (21–30 Hz) band in the somatomotor A region. At-rest recordings revealed that the sensorimotor region on day 1 pre-vibrotactile coordinated reset (vCR) **(A)** (*M* = 0.079 ± 0.036) significantly decreased in high beta relative power after 3 months of vCR treatment **(B)** (*M* = 0.058 ± 0.025).

For cumulative effects, a paired-samples t-test demonstrated a significant effect for relative power within the high beta band (21–30 Hz) in the sensorimotor region [*t*(4) = 3.012, *p* = 0.030, *SD* = 0.015]. Specifically, relative high beta power pre-vCR (*M* = 0.079, *SD* = 0.036) significantly decreased after 3 months of vCR stimulation (*M* = 0.058, *SD* = 0.025; Figure 7). Additionally, theta power was trending [*t*(4) = −2.508, *p* = 0.066, *SD* = 0.0183], in which theta power at pre-vCR (*M* = 0.181, *SD* = 0.091) increased after 3 months of vCR therapy (*M* = 0.202, *SD* = 0.021).

### Study 2: The Impact of Prolonged Vibrotactile Coordinated Reset Therapy on Parkinson’s Disease Motor Symptoms

#### Patient 1

**FIGURE 8:**
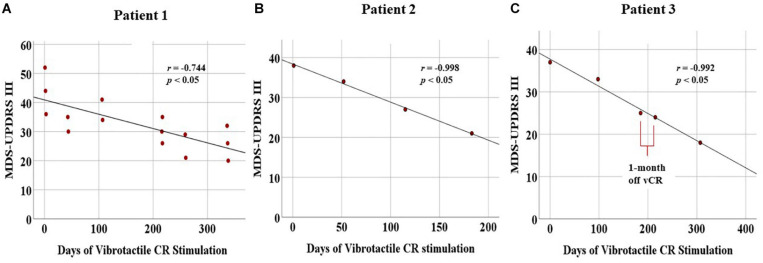
Displays cumulative chronic, months-long effects of vibrotactile coordinated reset (vCR) treatment. For all three patients, significant negative correlations for the Movement Disorders Society-Unified Parkinson’s Disease Rating Scale (MDS-UPDRS) III were found [Patient 1 **(A)**, *r* = –0.744, *p* = 0.001; Patient 2 **(B)**, *r* = –0.998, *p* = 0.002; Patient 3 **(C)**, *r* = –0.992, *p* = 0.001). Patient 3 **(C)** also exhibited a slight decrease in MDS-UPDRS III scores at the preplanned 1-month pause in stimulation between 6 and 7 months. These results suggest significant improvement of motor ability.

Sustained cumulative effects: Using Pearson’s correlation (two-tailed), we observed a significant linear decrease for the MDS-UPDRS III (*N* = 15, *r* = −0.744, *p* = 0.001) as well as subscores for tremor (*r* = −0.712, *p* = 0.003), rigidity (*r* = −0.660, *p* = 0.007), and bradykinesia (*r* = −0.671, *p* = 0.006). The axial subscore was trending (*r* = −0.492, *p* = 0.062; Figure 8A).

Acute effects assessed by difference scores (Delta MDS-UPDRS III): Overall, patient 1 exhibited greater acute decreases in MDS-UPDRS III scores in the beginning of treatment (baseline = -10) versus the last day of treatment (post-vCR = 0). For a detailed description of these difference scores, please see Supplementary Table 2.

Medication remained at a stable pre-vCR level in between visits (LEDD = 450 mg/day). Patient 1 remained at Hohn and Yahr (HY) scale 2 on medication (pre- and with vCR).

#### Patient 2

Sustained cumulative effects: Patient 2 displayed a linear decrease of his/her PD motor symptoms, as Pearson’s *r* correlations demonstrated a significant decrease in total MDS-UPDRS III scores (*N* = 4, *r* = −0.998, *p* = 0.002) as well as for the tremor subscores (*r* = −0.978, *p* = 0.022). Bradykinesia was trending in the same direction (*r* = −0.940, *p* = 0.060). Rigidity (*r* = −0.303, *p* = 0.697) and axial (*r* = −0.886, *p* = 0.114) were nonsignificant (Figure 8B).

Medication: From the onset of vCR, patient 2 had a reduction in medication use (LEDD decreased from 2,700 mg/day + 2–3 vapes of CBD/THC daily to LEDD 900 mg/day + 2–3 vapes of CBD/THC weekly). Although we did not prospectively collect PD off time diaries, subject 2 had > 90% reduction in self-reported off time despite LEDD reduction. The latter finding, however, must be handled with caution, since it was based on self-report. Patient 2 went from HY4 on medication (pre-vCR) to HY2 on medication (with vCR), while gait improved from using a cane consistently and wheelchair occasionally to walking without assistance (see Supplementary Video 2).

#### Patient 3

Sustained cumulative effects: Patient 3 underwent continuous improvement of his/her motor condition, as evidenced by Pearson’s *r* correlations demonstrating a significant decrease in the MDS-UPDRS III scores (*N* = 5, *r* = −0.992, *p* = 0.001) and the tremor subscores (*r* = −0.976, *p* = 0.005). No other significant changes were found (Figure 8C).

One-month long-term effects of vCR therapy: For the MDS-UPDRS III, difference scores obtained by subtracting the 6-month data from 7-month data revealed minimal differences (Delta MDS-UPDRS III = −1 (see Supplementary Table 3 for a detailed description of difference scores of the 1-month pause in stimulation).

Acute effects assessed by difference scores: Overall, patient 3 exhibited greater acute decreases in MDS-UPDRS III scores in the beginning of treatment (baseline = −10) versus the last day of treatment at 6 months (post vCR = −1). For a detailed description of all difference scores from baseline to every 3-month follow-up, see Supplementary Table 3.

From the onset of vCR, patient 3 reduced medications (LEDD from 920 mg/day at baseline to 820 mg/day at 10 months). At the start of treatment, Patient 3 went from HY3 off medication to HY2 off medication. Patient 3 had moderate postural instability at baseline to no impairment after 10 months of vCR treatment. Lastly, from months 7–10 of treatment, patient 3 was able to reduce his/her daily amount of vCR from 4 h to roughly 2 h three times per week.

On a final note, in Figure 5C, we visually compared baseline (*M* = 45, *SD* = 9.89) and 3-month (*M* = 34, *SD* = 9.89) MDS-UPDRS III data for the two patients in study 2 who received regular vCR to noisy vCR (Figure 5B).

### Computational Results

#### Vibrotactile Stimulation Modulates Neuronal Spiking Activity

**FIGURE 9:**
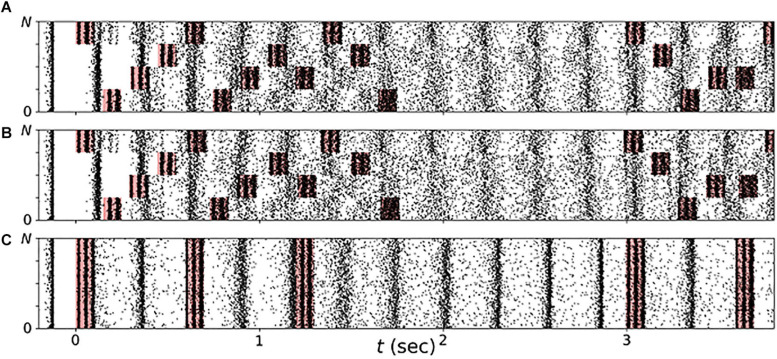
Stimulation shapes neuronal spiking activity. Raster plots of neuronal spiking activity during the first few seconds of vibrotactile stimulation with a regular vibrotactile coordinated reset (vCR) pattern **(A)**, a noisy vCR pattern (same sequence as regular CR but with jitter of 23.5%) **(B)**, and vibratory purely periodic multichannel stimulation (vPPMS) **(C)**. Sensory inputs caused by vibratory burst delivery to corresponding fingertips are marked red. Parameters: t = 0 marks onset of stimulation with *f*_CR_ = 1.67 *Hz* and *A* = *A*_0_.

We studied the response of our neuronal network model in the synchronized state to vibrotactile stimulation. Raster plots of representative spiking activity during onset of stimulation are shown in Figure 9 for regular vCR (Figure 9A) and noisy vCR (Figure 9B), as well as for vPPMS (Figure 9C). We found that stimulus delivery causes collective spiking events of the stimulated neuronal subpopulation. These were followed by a complex spike pattern, resulting from spiking events caused by excitatory input from other subpopulations, especially as they received stimuli, and time periods during which neurons were irresponsive to input because their membrane potentials were far from the spiking threshold. The combination of both typically resulted in phase shifts between collective spiking events of individual subpopulations and a broadening of the distribution of spike times during these events, especially during ON periods. In contrast, the collective rhythm remained intact during vPPMS, and only broadening of the distribution of spike times during collective spiking events was observed.

#### Long-Lasting Desynchronization by Vibrotactile Coordinated Reset Stimulation

We studied acute and long-lasting effects of noisy vCR stimulation in the neuronal network model. vCR was delivered for 1 h to networks in the synchronized state. To evaluate acute effects, the mean synaptic weight ⟨*w*⟩(*t*) and the Kuramoto order parameter ρ(*t*), Eq. (3), were calculated during stimulation. After cessation of stimulation, the simulation was continued to study long-lasting effects.

**FIGURE 10:**
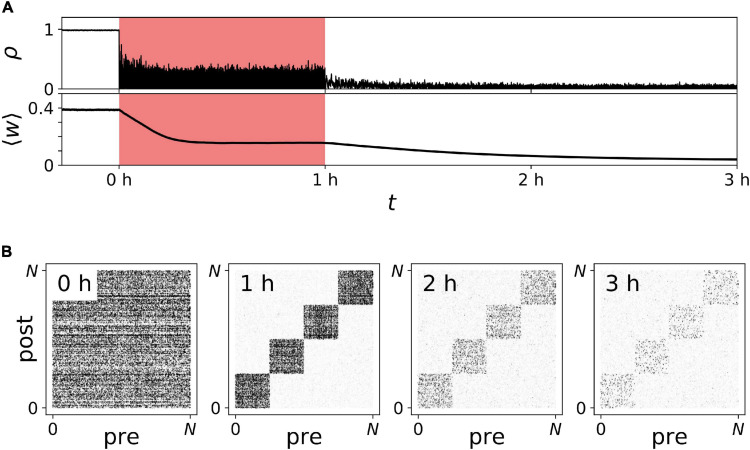
Long-lasting desynchronization by sensory stimulation with noisy vibrotactile coordinated reset (vCR). **(A)** Time trace of the Kuramoto order parameter ρ(*t*) and the mean synaptic weight ⟨*w*⟩(*t*) before, during (red), and after noisy vCR stimulation. **(B)** Snapshots of connectivity matrices containing the values of all synaptic weights *w*_i→j_(*t*) evaluated at indicated times after onset of stimulation; see also labels in panel **(A)**. Parameters: *J* = 23.5%, *f*_CR_ = 1.5 Hz, and *A* = *A*_0_.

Representative trajectories of ρ(*t*) and ⟨*w*⟩(*t*) before, during, and after noisy vCR stimulation are shown in Figure 10. We found that ρ(*t*) decreased during stimulation, demonstrating acute desynchronization in response to ongoing noisy vCR stimulation. Furthermore, ⟨*w*⟩(*t*) reduced gradually and finally approached a stationary value, indicating stimulation-induced decoupling of the neurons. After cessation of stimulation, ⟨*w*⟩(*t*) further decreased and the spiking remained desynchronized, indicating that stimulation drove the network into the basin of attraction of a stable desynchronized state.

In addition to the mean synaptic weight, Figure 10A, we analyzed the specific structure of the connectivity matrix in Figure 10B. As predicted in our previous work (Kromer and Tass, 2020), we found qualitative differences between the mean weight of synapses interconnecting different neuronal subpopulations and synapses connecting neurons in the same subpopulation (Figure 10B). In the following, these synapses are referred to as interpopulation and intrapopulation synapses, respectively. While interpopulation synapses weakened during stimulation, weights of intrapopulation synapses remained strong or even strengthened [Figure 10B (1 h)]. Thus, in the neuronal network model, noisy vCR stimulation mainly weakened interpopulation synapses. Nevertheless, stimulation led to long-lasting desynchronization (Figure 10A). Thus, weakening of interpopulation synapses was sufficient to drive the network into the basin of attraction of a stable desynchronized state.

#### Acute and Long-Lasting Effects of Vibrotactile Stimulation

**FIGURE 11:**
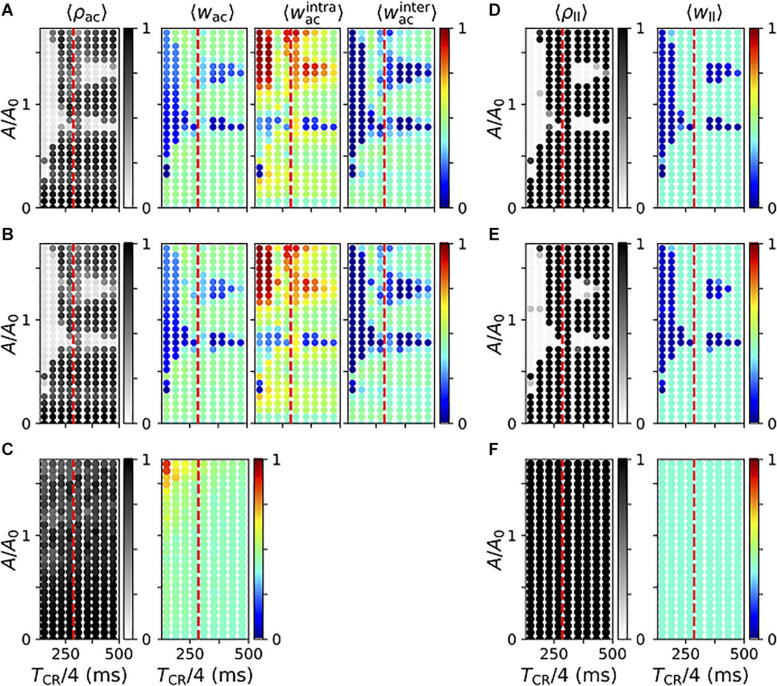
Parameter dependence of stimulation-induced desynchronization and weight dynamics in the neuronal network model. **(A–C)** Acute effects of regular vibrotactile coordinated reset (vCR) **(A)**, noisy vCR **(B)**, and vibratory purely periodic multichannel stimulation (vPPMS) **(C)**. Columns show results for the acute Kuramoto order parameter ρ_ac_ evaluated shortly before stimulation ceases (first column); the corresponding mean synaptic weight *w*_ac_ (second column); and mean weights of intrapopulation (third column) and interpopulation synapses (fourth column), respectively. **(D–F)** Corresponding long-lasting effects of regular vCR **(D)**, noisy vCR **(E)**, and vPPMS **(F)** evaluated 1 h after cessation of stimulation. Here, the first column shows the Kuramoto order parameter ρ_ll_ and the second column the mean synaptic weight *w*_ll_. For comparison, the mean period of the original synchronous rhythm (1/*f*_synch_,*f*_synch_ = 286 ms) is marked by vertical dashed red lines. All results were time-averaged over an interval of 12 s shortly before cessation of stimulation **(A–C)** and 1 h after cessation of stimulation **(D–F)**. Data points show ensemble averages (marked by angular brackets) over five network and sequence realizations. Results for noisy vCR were obtained using a jitter of 23.5%.

Next, we studied the parameter dependence of acute and long-lasting effects of vibrotactile stimulation in the neuronal network model. First, we considered the degree of acute synchronization as quantified by the Kuramoto order parameter, Eq. (3). Figure 11 displays results for regular vCR (**A**), noisy vCR (**B**), and vPPMS (**C**). We found that both vCR stimulation protocols caused pronounced acute desynchronization for sufficiently fast and strong stimulation and a wide range of stimulation amplitudes. In contrast, spiking remained synchronized during vPPMS.

In particular, we show the mean synaptic weight (second column) and the mean weights of intrapopulation (third column) and interpopulation synapses (fourth column) in Figure 11. Note that we did not distinguish between the latter two for vPPMS, as all neurons received stimuli simultaneously. We found a considerable decrease of the mean synaptic weight in the parameter regions where stimulation led to acute desynchronization. We further found that CR stimulation for which *T*_CR_/4 is smaller than the period of the synchronous rhythm, 1/*f*_synch_, caused more robust weight reduction with respect to the mean input firing rate *A*. For slower stimulation, weight reduction was limited to certain values of *A*. In contrast, vPPMS did not weaken synapses, instead it might even increase the mean synaptic weight for fast and strong stimulation, see Figure 11C.

We found qualitative differences between the dynamics of intrapopulation and interpopulation synapses during vCR stimulation. Intrapopulation synapses only weakened in a small portion of the parameter space, mostly for a well-defined mean input firing rate *A* (Figures 11A,B). In the remaining part, intrapopulation weights typically increased. Thus, vCR stimulation strengthened synaptic connections between neurons responding to the same vibrotactile bursts. In consequence, decoupling was mainly driven by a stimulation-induced reduction of interpopulation synapses. The latter occurred primarily in parameter regions where acute desynchronization was observed.

We found poor performance of vCR stimulation when *T*_CR_/4 was close to the inverse frequency of the synchronous rhythm, 1/*f*_synch_. In that case, stimulation stabilized the collective rhythm rather than inducing phase shifts between rhythms of individual neuronal subpopulations.

Too slow and/or weak stimulation was not capable of shifting spiking rhythms of separately stimulated subpopulations against each other reliably and caused only weak decoupling. On the other hand, too strong stimulation caused high firing rates during stimulus delivery. Then, the stimulated subpopulation provided strong excitatory input to the others, and their spiking rhythms aligned. Furthermore, strong stimulation caused a strengthening of intrapopulation weights. For intermediate stimulation amplitudes (*A* of the order of *A*_*0*_), we found a reliable reduction of the mean synaptic weight for a broad range of interstimulus intervals.

Next, we considered long-lasting effects of the different stimulation protocols. Simulation results for regular and noisy vCR stimulation are presented in Figures 11D,E. We found that regular vCR (Figure 11D) and noisy vCR (Figure 11E) showed pronounced long-lasting effects in parameter regions, where acute desynchronization was found (compare Figure 11D to Figure 11A and Figure 11E to Figure 11B, respectively). In contrast, vPPMS did not entail long-lasting desynchronization or changes of the mean synaptic weight (Figure 11F).

## Discussion

We studied the effects of vCR stimulation on PD patients in two clinical feasibility studies and in a computational neuronal network model with STDP. In the following, we discuss the results of our clinical studies and compare them to the effects of vibrotactile stimulation in the computational model.

### Study 1: The Impact of Noisy Vibrotactile Coordinated Reset on Motor and Cortical Beta Power Changes

Acute decreases in the MDS-UPDRS III after vCR treatment were mainly seen on day 1. At month 3, acute decreases in the MDS-UPDRS III after vCR treatment were only significant for tremor subscores. This may suggest that acute effects reduce over time, which is expected as the brain adapts to a more normalized state. MDS-UPDRS III scores significantly decreased after 3 months of vCR treatment. This finding may suggest that vCR motor improvement effects can be long lasting. Lastly, after 3 months of vCR treatment, patients showed no significant changes in the MDS-UPDRS I, II, and IV, PDQ-39, or LEDD compared to the first study visit. However, percentage decreases indicated that LEDD decreased by 7.82% on average. This is important, as it suggests that patients may decrease their LEDD when receiving vCR treatment, which in turn may provide substantial therapeutic relief from dopaminergic side effects. A greater sample size in future studies will address this finding further.

Acute relative EEG power vCR effects were not significant on the first visit or at the 3-month follow-up visit. Considering acute effects on the first visit were seen in MDS-UPDRS III scores after 2 h of stimulation, 10 min of vCR stimulation during the EEG recording may be an insufficient amount of time to cause significant cortical changes. Indeed, CR is a cumulative treatment, in which effects become greater after a sufficient amount of time (Hauptmann and Tass, 2009; Adamchic et al., 2014).

High beta band relative power (21–30 Hz) significantly decreased over the sensorimotor cortex after 3 months of vCR treatment. Studies have shown that PD patients display beta band coherence between motor cortical EEG areas and the STN (Marsden et al., 2001; Williams et al., 2002). Attenuation of synchronous beta activity in the STN occurs while PD patients are under the influence of L-dopa (Brown et al., 2001; Weinberger et al., 2006; Giannicola et al., 2010) or when DBS is delivered to the STN (Bronte-Stewart et al., 2009; Whitmer et al., 2012). Previous studies have suggested that the beta band can be subdivided into low and high bands, with low beta band activity decreasing in the STN when PD patients receive dopaminergic therapy (Priori et al., 2004; López-Azcárate et al., 2010). In addition, the high beta band has been associated mainly with gait activity, with increases in power relating to impaired freezing of gait (FoG) in cortical EEG motor areas (Ly et al., 2016) and in the STN (Toledo et al., 2014). Low beta activity has been related to bradykinesia and rigidity in the STN (López-Azcárate et al., 2010). While our sample size is too small to make significant claims regarding subtypes of PD, the relationship between the beta band power decrease in sensorimotor cortex after 3 months of vCR treatment is promising, as it suggests vCR therapy may modulate power in this band.

### Study 2: The Impact of Prolonged Vibrotactile Coordinated Reset Therapy on Parkinson’s Disease Motor Symptoms

All patients showed significant cumulative improvement in the MDS-UPDRS III. Patients 1 and 3 showed greater acute effects of vCR treatment in the beginning of the study compared to later follow-up visits. For LEDD amount, no patient increased his/her medication throughout the study. Patient 1 was able to maintain his/her medication and continuously improve motor ability. Patients 2 and 3 experienced a reduction in medication while constantly improving their motor scores. For patient 2, we also considered that patient 2 may have had “supra-on state FoG” (Espay et al., 2012) with improvement in FoG after vCR related to reduction of LEDD. However, patient and spouse reported that when FoG occurred, it reliably improved by taking short-acting L-dopa, arguing against this. Taking additional doses did not result in reoccurrence of FoG, but in dyskinesia. Patient 3 showed no notable changes in MDS-UPDRS III scores when comparing the 6-month data to the data at 7 months, i.e., after a preplanned 1-month pause in vCR stimulation. Additionally, this patient reduced his/her daily 4 h of vCR time to approximately 2 h 3 times a week after the 7-month follow-up and still showed exceptional improvements in the MDS-UPDRS III scores, as displayed in Figure 8C.

The cumulative decrease of off-medication MDS-UPDRS III scores observed in this study is remarkable since in PD patients, MDS-UPDRS motor scores typically increase over time. For instance, in a study in 362 patients with *de novo* PD, a linear increase of MDS-UPDRS scores was observed over 5 years, with an estimated 4.9 increase of total MDS-UPDRS per year (Holden et al., 2018).

Overall, in both studies, vCR was easily managed, yielded no side effects, and delivered significant cumulative and sustained improvement in the MDS-UPDRS III scores.

### Vibratory Displacement Effects

The first-in-human study using 0.35-mm vibration amplitude found no significant differences in the UPDRS III during the 3-day treatment phase (Syrkin−Nikolau et al., 2018). However, items for rigidity and speech were excluded in this study, which makes comparing our results difficult. Nevertheless, our finding of strong acute decreases in the MDS-UPDRS III observed during study 1’s first visit and in study 2 in patient 1’s first 3-day visit, and patient 3’s first day visit may indicate that smaller peak vibration amplitudes (0.1 mm/0.06 mm) are more beneficial toward treating patients’ motor symptoms.

The majority of mechanoreceptors of the glabrous skin of the human hand are fast adapting (FA), where FA I mechanoreceptors respond to 30–60-Hz vibrations, and FA II mechanoreceptors to 100–300 Hz (Johansson and Vallbo, 1983). Conduction velocities of FA I and FA II mechanoreceptors are in similar ranges (Knibestöl, 1973) but may still be different enough to compromise the vCR activation pattern. Smaller vibration amplitudes might be more beneficial for two reasons: (1) Smaller-amplitude 250-Hz vibrations might stimulate the FA II mechanoreceptors more selectively, which might be favorable in case of larger differences in conduction velocities of FA I and FA II mechanoreceptors; for details, see Tass (2017). (2) Smaller-amplitude 250-Hz vibrations of different fingertips might activate cortical representation areas with smaller spatial overlap, which is more favorable for CR stimulation; for details, see Tass (2017). In the computational model, we find better performance at moderate amplitudes as well. Here, high mean input firing rates corresponding to large amplitudes of skin indentation oscillations (Harvey et al., 2013) cause high firing rates of stimulated neurons during stimulus deliveries. Then, the stimulated subpopulation provides strong excitatory input to the other subpopulations that may result in synchronization of the spiking rhythms of separately stimulated subpopulations. Therefore, strong stimulation reduces phase shifts between the rhythms of individual subpopulations and reduces acute desynchronization effects.

### Limitations and Future Directions of Clinical Studies

Our clinical studies demonstrate encouraging therapeutic effects of chronic, months-long vCR therapy. Nevertheless, limiting factors prevent us from making strong conclusions regarding the efficacy of vCR to the general PD population because of a small sample size and lack of sham condition. However, we do not believe this treatment to be the result of a placebo effect for the following reasons: (1) It has been previously shown in PD patients that increases in motor function due to a placebo effect are longitudinally nonuniform, while our patients responded in a uniform way (Goetz et al., 2000). (2) Tremor is less susceptible to the placebo effect (Goetz et al., 2000). In our studies, study 1 showed trending cumulative decreases in tremor at 3 months of vCR treatment, and study 2 showed significant cumulative decreases in tremor for all patients. (3) In medical and surgical interventions, the overall positive placebo response rate using the MDS-UPDRS III is 16% (Goetz et al., 2008b). Our percentage decrease in MDS-UPDRS III at 3 months was 15.65%. However, given that vCR treatment is most effective when used for a longer period of time (Adamchic et al., 2014; Hauptmann and Tass, 2009), our 6+-month data may be a more reliable measure. For instance, when comparing MDS-UPDRS III baseline scores to the last day of treatment in study 2, the overall percentage decrease was 53.52%, over three times the expected placebo effect seen in long-term PD studies (Goetz et al., 2008b).

Study 1 was initially planned as a comparative 13-month study in 2 × 10 PD patients comparing the effects of regular vCR and noisy vCR (6 months of 2–4 h of regular vs. noisy vCR per day, followed by a preplanned 1-month vCR pause to assess whether vCR effects are long lasting, finally followed by 6 months of 2 h of regular vs. noisy CR three times per week). Due to COVID-19, only six patients in the noisy vCR group were able to finish the 3-month follow-up. Based on the clinical results presented here, we cannot infer outcome differences between regular vCR and noisy vCR, which is in agreement with our computational results presented.

### Computational Studies

We studied long-lasting effects of vibrotactile stimulation in a network of excitatory LIF neurons with STDP. Prior to stimulation, neuronal activity was synchronized. This model network represented a brain region expressing excessive neuronal synchrony during PD. Neurons were subject to excitatory input from a brain region with increased and periodically modulated spiking activity during vibrotactile stimulation. Delivery of vibratory burst stimuli resulted in a substantial increase and a periodic modulation of the input firing rate of this brain region, as observed experimentally in the human somatosensory thalamic nucleus (Weiss et al., 2009) and SI of macaque monkeys (Harvey et al., 2013). This affected pathological synchrony and caused desynchronization. Furthermore, it triggered a plastic reorganization of the network, which led to long-lasting changes of spiking activity. Future computational studies will be devoted to refining the modeling approach by taking into account more complex and physiologically more realistic network models that incorporate collective bursting (Powanwe and Longtin, 2019) and allow for studying the effect of stimulation on multiple coexisting pathological rhythms and cross-frequency coupling (Hyafil et al., 2015).

#### Comparison With Effects Caused by Electrical Model Stimuli

We found qualitatively different reshaping of intrapopulation and interpopulation synapses during application of regular and noisy vCR stimulation (Figures 11A,B). While intrapopulation synapses typically strengthened during stimulation, interpopulation synapses weakened. Weakening of interpopulation synapses was in accordance with previous results on electrical multisite CR stimulation presented in Kromer et al. (2020). However, results for intrapopulation synapses differed qualitatively. In more detail, Kromer et al. (2020) reported a weakening of intrapopulation synapses during electrical CR stimulation. This discrepancy may result from different statistics of neuronal spiking responses to administered stimuli. A recent theoretical study on networks of LIF neurons with STDP (Kromer and Tass, 2020) revealed two basic mechanisms for reshaping of network connectivity in response to spatiotemporal stimulus patterns: sequence and stimulus-induced reshaping. Sequence-induced reshaping describes synaptic reshaping as a consequence of the statistics of interstimulus intervals between stimuli administered to individual neuronal subpopulations. In contrast, stimulus-induced reshaping describes synaptic reshaping as a consequence of neuronal responses to individual stimuli. During CR stimulation, the former strongly affects interpopulation synapses, whereas the latter dominates the dynamics of intrapopulation synapses (Kromer and Tass, 2020). We expect that qualitatively similar results for interpopulation synapses during vCR and electrical CR stimulation were caused by the characteristic statistics of time lags in the CR pattern, used here and in Kromer et al. (2020). In contrast, individual stimuli used here and in Kromer et al. (2020) differed significantly. In Kromer et al. (2020), short, charged-balanced electrical pulses were used. These pulses resulted in a sharp collective spiking response of stimulated neurons. This led to a weakening of intrapopulation synapses by a delay-induced effect introduced as “decoupling through synchrony” by Lubenov and Siapas (2008). In contrast to short electrical stimuli, we modeled vibrotactile stimuli by a significant increase and modulation of the firing rate of presynaptic Poisson input. The distribution of spike times during individual collective spiking responses was rather broad and extended over tens of milliseconds (see raster plots in Figure 9). As discussed in Knoblauch et al. (2012), Kromer and Tass (2020), Lubenov and Siapas (2008), distributions that were broad compared to the axonal delays (3 ms in our model) led to synaptic potentiation, as presynaptic spikes often arrived before postsynaptic ones. This led to the strengthening of intrapopulation synapses in our neuronal network model (Figure 11). For a comparison with previously employed sensory model stimuli, we refer to Supplementary Discussion 3.1.

#### Comparison to Purely Periodic Multichannel Stimulation

We compared simulation results for regular and noisy vCR stimulation to those for vPPMS. Purely periodic multichannel stimulation (PPMS) was originally introduced in Zeitler and Tass (2018) and corresponded to the simultaneous stimulation of all four fingertips at the beginning of the CR cycles in a 3:2 ON-OFF pattern. Zeitler and Tass (2018) delivered PPMS at the frequency of the dominant synchronous rhythm using model stimuli that represented excitatory postsynaptic potentials, i.e., similar to Popovych and Tass (2012), as described in Supplementary Discussion 3.1. Synaptic weakening during PPMS was significantly weaker than during CR stimulation. They also reported entrainment between stimuli and spiking rhythms. In our network model, vPPMS neither caused acute nor long-lasting desynchronization.

The poor performance of vPPMS as compared to vCR stimulation indicates that separate stimulation of different neuronal subpopulations is critical for desynchronization. The latter corresponds to the originally proposed desynchronization mechanism of CR stimulation (Tass, 2003). vPPMS, however, did not destabilize the pathological synchronous rhythm but caused an entrainment with the stimulus pattern (Figure 9).

We found that the distribution of spike times during collective spiking events broadened during stimulation ON periods of vPPMS, whereas it sharpened again during stimulation OFF periods due to synaptic interactions (Figure 9). We speculate that uninterrupted vPPMS, i.e., delivered without OFF periods, may cause desynchronization by further broadening collective spiking events until these events disappear and the system desynchronizes.

vPPMS did not reduce synaptic weights. Instead, we found a slight increase of the mean synaptic weight in most parts of the parameter space (Figure 11C). This resulted from broad distributions of spike times during collective spiking events; see above. For strong vPPMS, synaptic weights increased substantially (Figure 11C). This increase was not observed by Zeitler and Tass (2018) probably because they considered short phase resetting stimuli; see previous paragraph.

The comparison of our computational results to those of previous studies (Popovych and Tass, 2012; Zeitler and Tass, 2018; Kromer et al., 2020; Kromer and Tass, 2020) indicates that the statistics of spiking responses to individual stimuli may have a strong impact on the outcome of stimulation. While model stimuli of previous studies led to a phase reset (Popovych and Tass, 2012; Zeitler and Tass, 2018; Kromer et al., 2020; Kromer and Tass, 2020), our vibrotactile model stimuli caused multiple collective spiking events and did not necessarily result in a phase reset of the collective rhythm. Nevertheless, vCR stimulation yielded pronounced acute and long-lasting desynchronization effects. This provides evidence that desynchronization effects of CR stimulation may not rely on phase-resetting stimuli and be more robust as originally assumed. In future studies, we intend to study the impact of the stimulus type on long-lasting desynchronization effects in more detail.

### Comparison Between Clinical and Computational Results

In both our clinical and our computational studies, vCR stimulation entailed pronounced long-lasting desynchronization of neuronal activity.

#### Measures of Synchrony

In our clinical studies, neuronal synchrony was quantified using EEG data of the sensorimotor cortex. High EEG power is typically associated with synchronous activity of a large population of neurons in the corresponding frequency band (Musall et al., 2014). However, it is generally assumed that the EEG is generated by postsynaptic potentials in neurons near the cortical surface rather than action potentials (Ebersole and Milton, 2003).

In our computational study, we measured neuronal synchrony using the Kuramoto order parameter. The latter measures the degree of in-phase synchronized spiking activity. Low values of the Kuramoto order parameter can correspond either to desynchronized neuronal activity or to partially synchronized states, such as cluster states (Tass, 1999). Our computational model possessed a rather limited set of stable dynamical states, i.e., desynchronized and synchronized states, that could be adequately distinguished using the Kuramoto order parameter. However, the latter may not be sufficient to distinguish between more complex dynamical states, such as multiple interacting rhythms (Hyafil et al., 2015) or chimera states comprising portions of synchronized and desynchronized neurons (Majhi et al., 2019).

In PD patients, synchronized activity in different frequency bands is related to different symptoms. Synchronized activity in the theta band (3–10 Hz) has been associated with symptoms such as dyskinesia and tremor (Brown, 2003; Steigerwald et al., 2008; Tass et al., 2010; Contarino et al., 2012) and synchronized activity in the beta band (13–30 Hz) with rigidity and bradykinesia (Kühn et al., 2006; Weinberger et al., 2006). Therefore, it is likely that different rhythms interact in PD-related brain networks. However, it is unclear whether chimera states occur in PD-related brain networks. In future studies, we anticipate considering more complex network models that allow us to study the influence of vibrotactile stimulation on networks with pathological synchrony in different frequency bands.

#### Effects of Vibrotactile Coordinated Reset Stimulation Build Up Slowly

In both our clinical and computational studies, effects of vCR stimulation built up slowly. In clinical study 1, we observed a significant improvement of the MDS-UPDRS III after 2 h of stimulation and a drop in high beta power over the sensorimotor cortex after 3 months of daily vCR stimulation, whereas no significant decrease of beta power was observed during and shortly after a 10-min vCR stimulation session during the first visit. This slow onset of stimulation effects was in marked contrast to classical DBS, where symptoms are suppressed within seconds to minutes after stimulation onset and resembled the slow buildup of effects observed in CR-DBS delivered to the subthalamic nucleus in PD patients (Adamchic et al., 2014). In our computational model, we observed an immediate reduction of the Kuramoto order parameter, corresponding to a reduction of neuronal synchrony (Figure 10). On the other hand, stimulation only slowly reduced synaptic weights. However, a sufficient reduction of the latter is required to drive the network into the attractor of a stable desynchronized and cause long-lasting desynchronization effects that outlast stimulation. Thus, while our model predicted a reduction of neuronal synchrony shortly after stimulation onset, which we did not observe in our clinical study, these effects only persisted after cessation of stimulation if a sufficient reduction of synaptic weights was achieved.

#### Regular Coordinated Reset and Noisy Coordinated Reset Lead to Similar Results

Neither our clinical nor our computational studies revealed qualitative differences between effects of regular and noisy vCR stimulation. While the number of patients considered in our clinical feasibility studies was too small to draw strong conclusions, our computational results created promising evidence that the effects of vCR stimulation are robust with respect to the considered stochastic variation of stimulus-onset times. In our neuronal network model, both regular and noisy vCR stimulation had pronounced acute and long-lasting desynchronization effects. These effects were most pronounced if more than one stimulus per cycle of the synchronous rhythm was delivered and stimulation was strong enough to drive neurons over the spiking threshold, such that it provides control over the neuronal spiking activity. Regular and noisy CR may have similar effects due to variable neuronal responses to vibrotactile stimuli. Unlike a sharp phase reset, vibrotactile stimuli caused several variable collective spiking events of the stimulated neuronal subpopulations. This might have masked the additional stimulus delivery time jitter, which was constrained to avoid vibrotactile masking of subsequent stimuli (Hollins et al., 1990; Figure 9). Note that vPPMS caused neither acute nor long-lasting desynchronization (Figure 11), which indicates that separate stimulation of neuronal subpopulations is essential for desynchronization effects.

#### Limitations and Future Directions

An interesting prediction of our computational model is that vCR predominantly weakens synapses that interconnect neuronal subpopulation that respond to stimuli delivered to different fingertips. It is however unclear how synapses between stimulated and not stimulated subpopulations are affected. In our clinical study, we observed a reduction of beta power over a wide region of the sensorimotor cortex (Figure 7) and a reduction of the MDS-UPDRS III, indicating that therapeutic effects of vCR stimulation were not limited to brain regions responding to vibrotactile stimulation of the fingertips. In order to understand the widespread therapeutic effects of vCR fingertip stimulation, we intend to address the propagation of desynchronization effects between different neuronal subnetworks in future studies.

### Proprioceptive Path to Motor Circuits and Beyond

During stimulation, vibrotactile bursts were delivered to the fingertips. Following the somatosensory pathway, high-frequency sinusoidal vibratory stimuli evoked strong spiking responses of thalamic neurons in the somatosensory thalamic nucleus [Vc (ventral caudal)] that are related to fast-adapting Pacinian corpuscle (PC) mechanoreceptors (Weiss et al., 2009). Furthermore, a substantial increase in the firing rates and pronounced phase locking of neuronal activity to vibratory stimuli was observed. Tactile Vc neurons relay sensory input to the somatosensory cortex SI. Experiments in macaque monkeys showed that sinusoidal high-frequency vibrotactile stimulation causes phase-locked spiking responses of SI neurons (Harvey et al., 2013). The authors also observed a logarithmic increase of the neurons’ mean firing rate with the stimulation amplitude.

The complex interconnectivity of cortical areas is to date not completely understood. It is therefore not known how cortical activity patterns in SI propagate to motor areas exhibiting pathological oscillations in PD (Lindenbach and Bishop, 2013). It is possible that propagation occurs *via* projections from somatosensory cortical areas to the motor cortex. Such connections have been observed in mammals across species (Jones and Powell, 1968; Jones and Powell, 1969; Mao et al., 2011). Furthermore, experimental studies reported that some thalamic neurons in the cat sensory nucleus ventralis posterolateralis and corresponding nuclei in monkeys project directly to the motor cortex [see Asanuma and Mackel (1989) for a review].

We found long-lasting changes of cortical activity in PD patients after vCR stimulation (Figure 7). Previous studies reported a long-lasting reorganization of motor areas in response to tetanic stimulation of the somatosensory cortex (Iriki et al., 1989; Keller et al., 1990). Another study reported that sensory stimulation of the pharynx also evokes long-lasting changes of excitability and the organization of the swallowing motor cortex (Hamdy et al., 1998). These changes were shown to outlast stimulation for at least 30 min, indicating that sensory stimulation can cause long-lasting changes in the motor cortex.

vCR stimulation may also activate proprioceptive pathways. Proprioceptive feedback is, for instance, provided by both muscle spindle afferents (Goodwin et al., 1972) and cutaneous receptors in the skin (Edin and Abbs, 1991; Collins et al., 2005). The frequency response of muscle-sensitive proprioceptive receptors is typically limited to approximately 120 Hz (Burke et al., 1976; Roll and Vedel, 1982) but may rise to 220 Hz for the most sensitive receptors (Burke et al., 1976; Roll et al., 1989). However, the frequency response of cutaneous receptors can be as high as 280 Hz (Ribot-Ciscar et al., 1989). In our clinical studies presented here, we used vibratory frequencies of 250 Hz, which may have led to a predominant activation of cutaneous receptors. Note, Lee et al. (2012) show results at 250 Hz with C-2 tactors (i.e., very similar mechanical stimulators used in this study) that indicate proprioceptive receptor response (postural).

Proprioceptive feedback during voluntary movements has been found to diminish parkinsonian tremor (Naros et al., 2018). Furthermore, thalamic neurons in the ventral intermediate nucleus (Vim), a target area for chronic deep brain stimulation as a treatment of tremor-dominant PD (Benabid et al., 1996), respond to proprioceptive stimulation (El-Tahawy et al., 2004). Evaluating tremor suppression for different electrode locations within the Vim, Milosevic et al. (2018) found that stimulation sites near the border between the Vim and the Vc lead to most efficient tremor reduction. Neurons in that border region respond to somatic inputs arising in muscle, joints, and deep tissue (Vitek et al., 1994; El-Tahawy et al., 2004). Milosevic et al. (2018) suggested that the stimulation-induced interruption of the pacing of proprioceptive input may contribute to tremor suppression.

Diminished amounts of brain-derived neurotrophic factor (BDNF) have been found in cerebrospinal fluid (CSF) within the ventricular or lumbar regions, autopsy reports of PD patient brains, and in Parkinson disease-induced [1-methyl-4-phenyl-1,2,3,6-tetrahydropyridine (MPTP) and 6-hydroxydopamine (6-OHDA)] animal models (Nagatsu and Sawada, 2005). This decrease in BDNF has been associated with dopamine depletion in the substantia nigra pars compacta and destruction of presynaptic terminals in addition to dopamine shortages that account for the majority of movement abnormalities in PD individuals (Nagatsu et al., 2000). BDNF is an abundant “neuroprotectant” protein found in the hippocampus and cerebral cortices (Deogracias et al., 2012). BDNF is delivered to the striatal area from the hippocampus and cerebral cortex (i.e., afferent pathway; Baquet et al., 2004). This nerve growth factor enhances growth of all affected neurons in PD (Zuccato and Cattaneo, 2007). Vibrational stimulation likely utilizes a similar pathway and may provide therapeutic PD benefits *via* upregulating BDNF resulting in increases in dopaminergic neurons. This hypothesis has been tested in a previous study of MPTP-treated mice showing that 4 weeks of low-amplitude (5 mm) vibrational therapy (where mice were freely roaming on a vibrational platform) significantly increased the amount of nigrostriatal dopaminergic neurons and quantity of BDNF (Zhao et al., 2014). This may suggest that vibrational therapy could have a beneficial dopaminergic effect on the nigrostriatal pathway within the PD brain. One might hypothesize that roaming-related sequences of vibrational bursts could exert desynchronizing effects on motor circuits. In that case, disease-modifying effects of vibrational therapy might possibly resemble those of STN-DBS observed in several animal studies (Chen et al., 2000; Maesawa et al., 2004; Wallace et al., 2007; Khaindrava et al., 2011; Spieles-Engemann et al., 2011; Musacchio et al., 2017), e.g., attributed to a reduction of glutamatergic excitotoxicity of the substantia nigra pars compacta (Chen et al., 2000; Maesawa et al., 2004; Nakao et al., 1999; Piallat et al., 1996; Wallace et al., 2007).

## Conclusion

Our clinical and computational results illustrate promising therapeutic effects of vCR therapy. Clinically, PD patients exhibited sustained cumulative benefits from continued used of vCR treatment. In conclusion, the results presented here demonstrate the feasibility and preliminary efficacy of vCR, which will properly enable us to plan a proof-of-concept study.

## Data Availability Statement

The original contributions presented in the study are included in the article/Supplementary Material, further inquiries can be directed to the corresponding author.

## Ethics Statement

The studies involving human participants were reviewed and approved by Stanford Institutional Review Board. The patients/participants provided their written informed consent to participate in this study. Written informed consent was obtained from the individuals for the publication of any potentially identifiable images or data included in this article.

## Author Contributions

JK and PT designed the computational study and analyzed computational data. JK performed the simulations. KP, RD, CH, and PT designed the clinical study protocol. KP, AC, TH, EL, AF, CH, and PT performed the clinical exams and/or took care of patients. KP, SH, and PT contributed to clinical data analysis. KP and AC performed EEG recordings. KP, AC, and PT analyzed the EEG data. BM and PT designed the vibrotactile glove system. KP, JK, and PT wrote the manuscript. All authors revised/approved the manuscript.

## Conflict of Interest

TH works as consultant for Boston Scientific and received teaching honoraria for DBS courses. RD has served as a clinical trials investigator for Impax Pharmaceuticals, Pharma2B, CALA Health, Axovant and Neurocrine Biosciences. CH has received speaking honoraria and consulting fees from Boston Scientific, Medtronic, and NeuroPace. PT works as consultant for Boston Scientific Neuromodulation and Gretap AG and is inventor on a number of patents for non-invasive neuromodulation. BM is employed by Engineering Acoustics who manufacture vibrotactile systems. No further disclosures. The remaining authors declare that the research was conducted in the absence of any commercial or financial relationships that could be construed as a potential conflict of interest.
