# Supplementary material for: Coordinated Reset Vibrotactile Stimulation Induces Sustained Cumulative Benefits in Parkinson’s Disease
Source: Front Physiol. 2021 Apr 6;12:624317. doi: 10.3389/fphys.2021.624317 (PMC8055937; doi:10.3389/fphys.2021.624317)
Supplement: Supplementary file 3 [file Data_Sheet_1.pdf]

Supplementary Material

1 Supplementary Vibrotactile Glove Material

Touch comprises multiple distinct systems, collectively known as the somatosensory system. Various sensory receptors are located in the skin (dermis and epidermis) as well as in the muscles, tendons and joints. These provide us with information regarding light touch, vibration, pressure, temperature, proprioception (position sense) and pain (Lederman and Klatzky, 2009).

Sensory receptors are concentrated in areas of the body that are associated with exploration (Cholewiak and Collins 1991). These areas are highly sensitive to mechanical stimuli. The organization of the somatosensory cortex shows a similar scaling, known as the cortical homunculus.

1.1 Physiological design

The skin and the underlying anatomical structure differ over the body. The skin is viscoelastic with a mechanical impedance that is complex (Edwards and Marks, 1995). The mechanical impedance is also influenced by the contact area and its static indentation.

Mechanical energy can produce shearing forces in the skin that dissipate with a distance from the source according to an inverse square law (Cholewiak and Collins 1991). However, vibration can also generate traveling waves of energy across the surface of the skin (Franke, 1951) and these can also be transmitted over large distances (Cholewiak and Collins 1991). For example, a stimulus on the finger can excite a greater number of distant receptors unless a damper, such as a static ring, is used around the stimulus site to damp surface waves.

Different types of sensory nerve endings are located within the skin; shallow receptors in the dermis include Merkel's disks, Ruffini cylinders, and Meissner's corpuscles, while deeper receptors include Pacinian corpuscles. The interaction between the mechanical properties of the skin and tactile sensory perception can be modeled (Phillips and Johnson, 1981). The displacement of a surface against the skin can be used to define the stimulus together with the contact characteristics and the dynamics. For vibrotactile excitation, a relatively simple relationship between the volume displacement and sensory stimulus can be used as a measure of sensory stimulus.

1.2 Actuator design

Vibrotactile actuators, or tactors, can be classified with respect to their ground reference. Actuators are attached to a large external mass and their ground reference corresponds to the external support. In

contrast, wearable actuators are lightweight, rest on the skin and thus have a ground reference that is internal to the body.

Our design approach was to configure our tactors to have a "contactor" (*C*) that oscillates perpendicularly to the skin, surrounded by a housing (*H*) and radial gap. The moving "contactor" was lightly preloaded against the skin (Zv_c), as well as the housing (Zv_H). When an electrical signal was applied (producing a force *F*), the "contactor" oscillated with a velocity (V_c) perpendicular to the skin, while the surrounding skin area was "shielded" with a passive housing (Mortimer et al., 2007). This provided a strong, point-like sensation that is easily felt and localized. The equivalent mechanical impedance for the skin load together with the free-body diagram for a wearable tactor is shown in **Figure S1**.

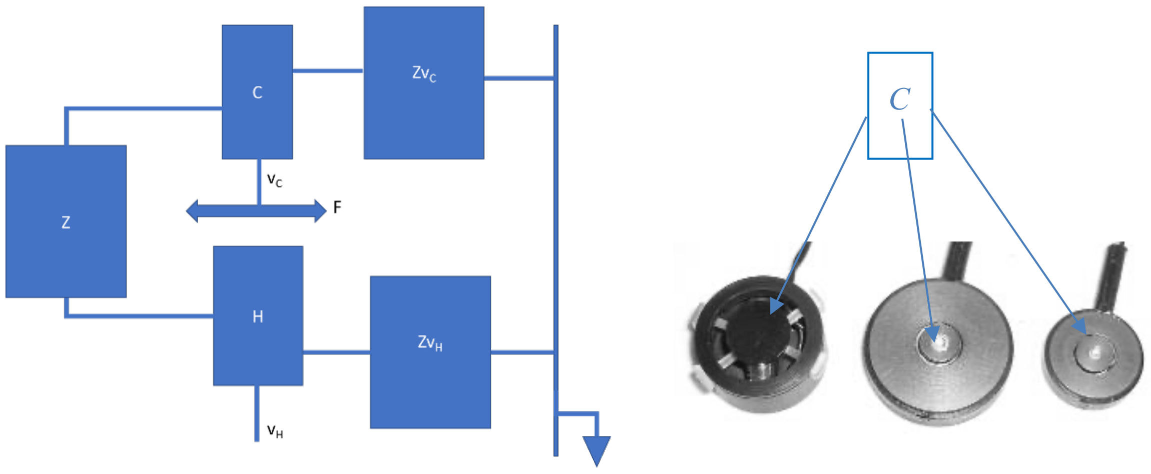

Figure S1 Engineering Acoustics, Inc (EAI) approach to the design of vibrotactile transducers; free-body diagram (left) and underlying tissue (top right), the EMR, C-2 and C-3 tactors with a contactor and front housing face separated by a radial gap (bottom right).

In this diagram the velocity of the housing is represented by V_H, the contactor velocity is V_C, the mass of the tactor housing which contains the motor is *H*. The skin load impedance (Moore et al., 1972) on the contactor and housing comprises an equivalent mass, mechanical compliance, and mechanical resistance.

For a fixed frequency (and geometrical configuration), the mechanical impedance of the skin presented to the contactor depends only on the contactor diameter (Mortimer et al., 2007). Larger contactors would result in a larger volume displacement in the skin and potentially an increase in perceived vibrational stimulus, but the actuator will also require a higher drive force to achieve an equivalent

displacement. The relative masses and contact areas of the housing and contactor can also be used to impedance match the skin mechanical impedance.

We could estimate the vibrotactile system requirements that were needed for testing vibrotactile Coordinated Reset Fingertip Stimulation (vCR). The fingertips are the most sensitive areas for vibration, and each finger has similar sensitivities to vibration and is expressed on adjacent cortical columns in the primary somatosensory cortex (S1).

The dynamics and mechanics of interaction of a fingertip with a grating contribute to tactile perception (Jones and Lederman, 2006). Four separate populations of tactile afferents are known to be located in the fingertip (Vallbo and Johansson, 1989). The fingertips are most sensitive to vibration at frequencies between about 200 to 300 Hz. These frequencies excite primarily the Pacinian corpuscles which are rapidly adapting (RA) receptors.

Reasonable technical requirements for the vibrotactile actuator for vCR would therefore be an operating frequency of approximately 250 Hz, a displacement output that exceeds 40 dB (Re 1μm) above the threshold for sensitivity for the fingers (to achieve salience), and a rise time of less than 2 ms.

The C-MF tactor (Engineering Acoustics, Inc.) had been specially designed for use on the fingers. The C-MF had a smaller contactor than the C-2 to optimize the impedance transfer between the actuator and finger. The C-MF housing is specially shaped to allow accurate and consistent finger mounting that is relatively unaffected by loading. The C-MF is shown in **Figures S2 and S3**.

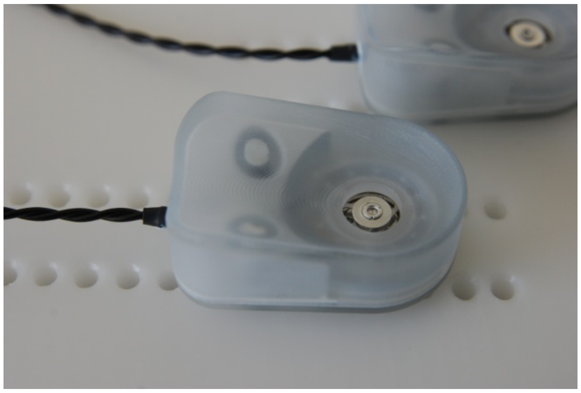

Figure S2 C-MF tactor designed for vCR fingertip stimulation.

1.3 Gloves

The hand array comprised of 8 C-MF tactors in two groups of 4. Each tactor was located against the fingertip of the second (index), third, fourth and fifth (pinky) finger using individual finger pods that

are attached to a glove. The pod isolated each finger from adjacent digits using a compliant fabric and could be adjusted to the user's finger length.

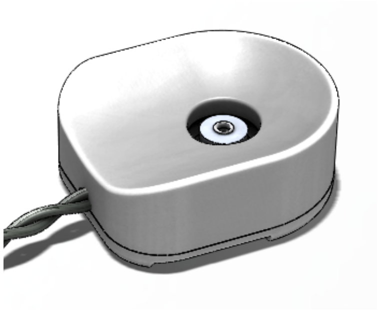

Figure S3 C-MF tactor designed for vCR fingertip stimulation.

2 Details on Computational Study

2.1 Neuronal Network Model

We considered a homogeneous network of $N = 1000$ excitatory LIF neurons with STDP. The probability for a connection between two randomly selected neurons was set to 7%. This was motivated by earlier studies on desynchronization of plastic neuronal networks by means of CR and RR stimulation (Kromer et al., 2020; Kromer and Tass, 2020).

The dynamics of the ith neuron's subthreshold membrane potential $V_i(t)$ followed from

$$C_i \frac{d}{dt} V_i(t) = g_L(V_{rest} - V_i(t)) + g_{syn,i}(t)\big(V_{syn} - V_i(t)\big) + I_{input,i}(t). \tag{S1}$$

C_i is the membrane capacitance, g_L the leak conductance, V_{rest} the resting potential, $g_{syn,i}$(t) the time-dependent synaptic conductance, V_{syn} the synaptic reversal potential, and $I_{input,i}(t)$ input from other brain regions. If $V_i(t)$ crossed the dynamic threshold potential $V_{th,i}(t)$, an artificial spike was added by setting $V_i(t)$ to V_{spike} for a time period of τ_{spike}. Afterwards, a reset was performed: $V_i(t) \rightarrow V_{reset}$ and $V_{th,i}(t) \rightarrow V_{th,reset}$. Otherwise, $V_{th,i}(t)$ obeyed the dynamics

$$\tau_{th} \frac{d}{dt} V_{th,i}(t) = V_{th,rest} - V_{th,i}(t). \tag{$S2$}$$

τ_{th} is the threshold time scale and $V_{th,rest}$ the resting threshold potential.

Neurons were connected via excitatory synapses. Dynamic synaptic conductances $g_{syn,i}(t)$ were updated when presynaptic spikes arrived. In particular, $g_{syn,i}(t)$ followed from

$$\tau_{syn}\frac{d}{dt}g_{syn,i}(t) = -g_{syn,i}(t) + \kappa\frac{\tau_{syn}}{N}\sum_{j\in G_i} w_{j\to i}(t)\sum_{l^j}\delta\left(t - t_{l^j}^j - t_d\right). \qquad (S3)$$

τ_{syn} is the synaptic time scale, κ the maximum coupling strength, and t_d the synaptic transmission delay. $w_{j\to i}(t) \in [0,1]$ are the time-dependent synaptic weights and scale the strengths of individual synapses. $\delta(t)$ is the Dirac delta distribution. The outer sum in Eq. (S3) runs over the set of neuron i's presynaptic neurons G_i. The inner sum runs over all spike times $t_{l^j}^j$ of the presynaptic neuron j.

Individual neurons were subject to noisy input $I_{input,i}(t)$. This was modelled by feeding Poisson spike trains into excitatory synapses on neuron i. $I_{input,i}(t)$ was obtained from

$$I_{input,i}(t) = g_{input,i}(t)\big(V_{syn} - V_i(t)\big),$$

$$\tau_{syn}\frac{d}{dt}g_{input,i}(t) = -g_{input,i}(t) + \kappa_{input}\tau_{syn}x_i(t). \qquad (S4)$$

κ_{input} scales the strength of the inhomogeneous Poisson input and $x_i(t)$ is the Poisson spike train fed into neuron i,

$$x_i(t) = \sum_{k_i}\delta\left(t - t_{k_i}\right). \qquad (S5)$$

The sum runs over all spike times t_{k_i} of the Poisson spike train fed into neuron i.

Spike trains $x_i(t)$ for the inhomogeneous Poisson input were generated according to the time-dependent firing rate $f_{input}(t) = f_{bg} + f_{vib}(t)$, see main text. The homogeneous part, f_{bg}, modeled stochastic background input from other brain regions and its firing rate was set to $f_{bg} = 20$ Hz (Kromer and Tass, 2020). The inhomogeneous contribution modeled input as a result of vibrotactile stimulation. Contributions to $f_{vib}(t)$ are described in the main text.

We used the parameter set: $g_{leak} = 0.02$ mS/cm^2 , $V_{rest} = -38$ mV, $V_{reset} = -67$ mV, $V_{th,reset} = 0$ mV, $V_{th,rest} = -40$ mV, $\tau_{th} = 5$ ms, $V_{syn} = 0$ mV, $\tau_{syn} = 1$ ms, $\tau_d = 3$ ms, $\kappa = 8$ mS/cm^2, $\kappa_{input} = 0.026$ mS/cm^2 (Kromer and Tass, 2020). In order to generate heterogeneity among the individual neurons' firing rates, we used Gaussian-distributed membrane capacitances C_i with mean value $\langle C_i\rangle = 3$ μF/cm^2 and standard deviation std$(C_i) = 0.05\,\langle C_i\rangle$.

The dynamics of the synaptic weights $w_{i\to j}(t)$ was determined by STDP (Bi and Poo, 1998; Markram et al. 1997). Weights were updated, $w_{i\to j}(t) \to w_{i\to j}(t) + W(\Delta t)$, whenever a postsynaptic neuron spikes, $t = t_{post}$, or a presynaptic spike arrived at the postsynaptic neuron, $t = t_{pre} + t_d$. Here, t_{pre} and t_{post} denote the spike times of the pre- and postsynaptic neurons, respectively. The STDP function $W(\Delta t)$ was modeled by two exponentials (Song et al., 2000)

$$W(\Delta t) = \eta \begin{cases} e^{-\frac{\Delta t}{\tau_+}}, & \Delta t > 0 \\ 0, & \Delta t = 0 \\ -\frac{\beta}{\tau_R} e^{-\frac{|\Delta t|}{\tau_-}}, & \Delta t < 0 \end{cases}. \quad (S6)$$

$\eta \ll 1$ scales the weight update per spike, τ_+ and $\tau_- = \tau_R \tau_+$ are the STDP decay times for synaptic potentiation and synaptic depression, respectively, and β is the ratio of overall synaptic long-term depression to long-term potentiation, $\Delta t = t_{post} - t_{pre} - t_d$ is the time lag between the current postsynaptic spike and the latest presynaptic spike arrival (when the weight update was triggered by a postsynaptic spiking event) or between the current presynaptic spike arrival time and the latest postsynaptic spike time (when the update was triggered by the arrival of a presynaptic spike at the postsynaptic neuron). This STDP scheme was used in previous studies on CR stimulation of plastic neuronal networks (Kromer et al., 2020; Kromer and Tass, 2020; Manos et al., 2018; Popovych and Tass, 2012). A detailed discussion of different STDP schemes can be found, for instance, in (Burkitt et al., 2004). In the main text, we used the STDP parameters $\eta = 0.02$, $\tau_+ = 10$ ms, $\tau_R = 4$, and $\beta = 1.4$ (Kromer et al., 2020; Kromer and Tass, 2020).

2.2 Scale of Stimulation Amplitude

In order to get a reasonable scale for A, we considered the average elevation of the membrane potential of an isolated LIF neuron due to vibrotactile stimulation causing the mean input firing rate A. For $A = A_0$ with $A_0 = \frac{(V_{th,reset} - V_{reset})\langle C_i \rangle}{(V_{syn} - V_{reset}) T \kappa_{input} \tau_{syn}} - f_{bg}$ the total postsynaptic current was sufficient to drive the neuron's membrane potential from V_{reset} to the maximal spiking threshold $V_{th,reset}$ neglecting the intrinsic dynamics. Therefore, such stimulation caused at least one spike during a vibratory burst of duration T. Note that the intrinsic dynamics and recurrent synaptic input may have led to more than one spike per burst.

2.3 Numerical Integration

Numerical integration was performed using the explicit Euler scheme with time step $h = 0.1$ ms. For the stochastic input, Eq. (S4), we used $\int_t^{t+h} dt' x_i(t') = N_i(t)$, where $N_i(t)$ is the stochastic spike count in the interval $[t, t+h]$. $N_i(t)$ was considered to be a Poisson random number with mean $f_{input}(t)h$.

2.4 Coexistence of Synchronized and Desynchronized States

In plastic networks of excitatory neurons, the set of coexisting stable states is determined by the STDP function, Eq. (S6), (Ocker et al., 2015). To study multistability in our computational model, we evaluated the Kuramoto order parameter, $\rho(t)$, and the mean synaptic weight, $\langle w \rangle(t)$, for different initial mean weights $\langle w \rangle(t = 0)$. The latter were realized by setting a fraction of weights to one, while the rest was set to zero. The average was taken over all synapses. Simulated trajectories of $\rho(t)$ and

$\langle w \rangle(t)$ are shown in **Figure S4**. Networks that had been initialized with many strong synapses approached a synchronized state with $\langle w \rangle \approx 0.38$, see **Figure S4** (A and B). In contrast, networks that had been initialized with only a few strong synapses approached a desynchronized state with low mean weight (see **Figure S4** A and B). Raster plots of neuronal spiking activity in either state are shown in **Figure S4 (**C and D). In the synchronized state, the network possessed collective oscillations at a frequency of $f_{synch} \approx 3.5$ Hz. These results were in accordance with previous studies on inhomogeneous networks (Kromer et al., 2020; Kromer and Tass, 2020).

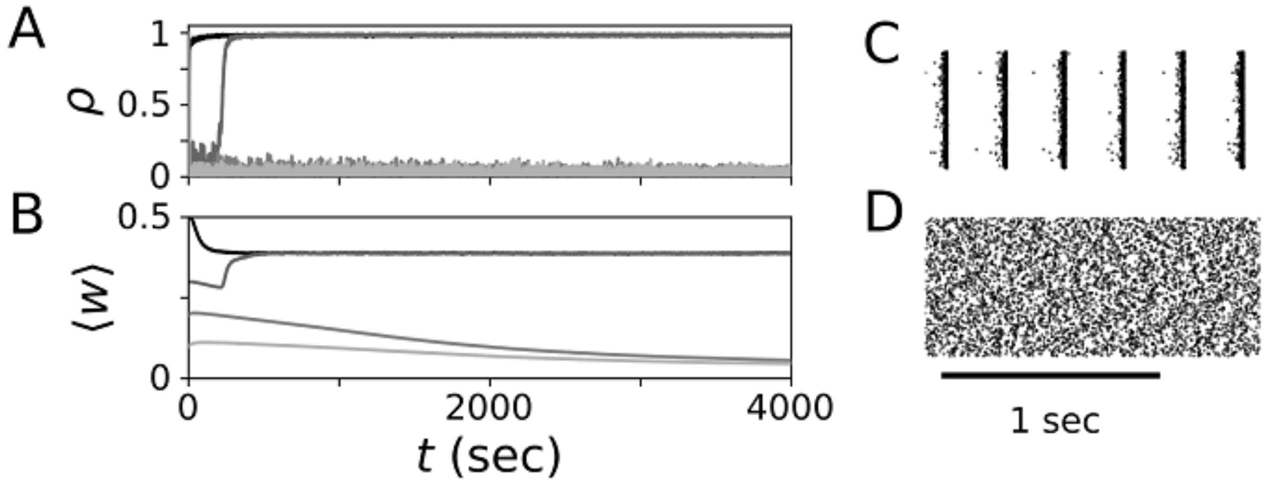

Figure S4 Coexistence of stable synchronized and desynchronized states in the neuronal network model. A: Kuramoto order parameter (assessing coincident firing), Eq. (3), for different initial mean weights. B: Corresponding trajectories of mean synaptic weights. Grayscale is the same as in panel A. Networks with high initial mean weight (dark) approached a stable synchronized state, while networks with low initial mean weight approached a stable desynchronized state (light). Representative raster plots of spiking activity in the synchronized and desynchronized state are shown in panels C and D, respectively. Parameters: Initial mean weights are $\langle w \rangle(t = 0) = 0.1, 0.2, 0.3,$ and 0.5 from bottom to top in panel B. Raster plots show spiking activity after a relaxation time of 3000 sec for $\langle w \rangle(t = 0) =$ 0.5 (C) and $\langle w \rangle(t = 0)$ =0.1 (D).

Networks considered in the main text were prepared in the synchronized state. This was done by setting $\langle w \rangle(t = 0) = 0.5$ and performing simulations for 3000 sec prior to stimulation so that the network reached the stationary synchronous state (see **Figure S4**).

3 Supplementary Discussion

3.1 Comparison with Previously Employed Sensory Model Stimuli

The qualitatively different results for the stimulation-induced dynamics of intrapopulation weights observed in our study and in (Kromer et al., 2020) suggest a strong impact of the shape of individual stimuli on the stimulation-induced weight dynamics. In a previous computational study, direct somatic CR stimulation was compared to synaptically mediated electrical CR stimulation, corresponding to the activation of incoming fibers (Popovych and Tass, 2012). The following stimuli were considered: (i) a burst of short charged-balanced electrical pulses delivered to the stimulated neuronal sub-population (direct stimulation), (ii) a single excitatory postsynaptic potential (incoming fiber of an excitatory neuron), and (iii) a single inhibitory postsynaptic potential (incoming fiber of an inhibitory neuron). Networks of spiking and bursting neurons interconnected by excitatory and inhibitory synapses were considered (Popovych and Tass, 2012). Stimuli were delivered in form of a regular 3:2 ON-OFF CR RVS pattern, corresponding to the regular vCR pattern in **Figure 1** A. The authors found that all three types of stimuli led to a phase reset and caused qualitatively similar desynchronization effects. They argued that the desynchronization effect of CR stimulation mainly relies on the phase resetting property of individual stimuli, i.e., whether the phases of simultaneously stimulated neurons align independently of their phases before stimulus delivery. Phase-resetting stimuli however cause an alignment of neuronal spike times, and thus, may result in rather sharp distributions of spike times during collective spiking events. They therefore lead to either synaptic weakening or strengthening of intrapopulation synapses depending on the length of synaptic transmission delays, see above. Note that Popovych and Tass did not consider synaptic transmission delays (Popovych and Tass, 2012). In contrast to the work of Popovych and Tass, the vibrotactile model stimuli considered here did not necessarily lead to a phase reset. Nevertheless, we found that regular vCR and noisy vCR stimulation caused acute and long-lasting desynchronization (see **Figure 11**).

4 Supplementary Tables

Table 1: Study 1 Patient Demographics

Gender	Age (years)	H & Y	TD/PIGD/I	SCOPA-COG	Baseline LEDD	3 Month LEDD
4 men<br>2 women	*M* = 53.33<br>*SD* = 10.78	*M* = 2.33<br>*SD* = 0.51	TD = 4<br>PIGD =1<br>I = 1	*M* = 32.16<br>*SD* = 2.99	*M* = 711.50<br>*SD* = 207.85	*M* = 644.83<br>*SD* = 229.85

M = Mean, *SD* = Standard Deviation, H & Y = Hoehn and Yahr stage, TD = Tremor Dominant classification, PIGD = postural instability/gait difficulty classification, I = Intermediate classification, SCOPA-COG = Scales for Outcomes in Parkinson's Disease-COGnition, LEDD = Levodopa Equivalent Daily Dose.

Table 2: Study 2 Patient 1 Acute Difference Scores

Month	MDS-UPDRS III Total	Tremor	Rigidity	Bradykinesia	Axial
Baseline	-10	-1	0	-7	-2
3 months	-8	0	-4	-3	-1
6 ½ months	-7	-3	-3	-1	0
10 months	0	1	0	0	-1

Table 2 describes the acute affects for patient 1 by subtracting pre-treatment vCR morning scores (≥ 8 hours without vCR) from post-treatment vCR motor scores (immediately after 4 hours of vCR) done at baseline and at approximately every 3-month visit. Based on these descriptive results, patient 1 exhibited greater acute decreases in MDS-UPDRS III scores in the beginning of treatment versus the last day of treatment.

Table 3: Study 2 Patient 3 Acute Difference Scores and 1 Month Pause in Stimulation Difference Scores

Month	MDS-UPDRS III Total	Tremor	Rigidity	Bradykinesia	Axial
Baseline	-10	-5	-3	-2	0
3 months	-21	-6	-1	-9	-5
6 months	-1	-3	2	0	0
7 months	5	0	2	4	-1
10 months	-1	-3	1	2	-1
6 vs 7 months (1 Pause in stimulation)	-1	0	2	-4	1

Table 3 describes the acute effects for patient 3. From baseline to 10 months, acute effects were calculated by subtracting pre-treatment vCR morning scores (≥ 8 hours without vCR) from post-treatment vCR motor scores (immediately after 4 hours of vCR). These acute results indicate that patient 3 demonstrated greater acute decreases in MDS-UPDRS III scores in the beginning of treatment versus the last day of treatment. Between the 6- and 7-month visit, patient 3 underwent a pre-planned 1-month pause in stimulation. To understand long term effects of vCR during the 1-month pause, we subtracted 6-month MDS-UPDRS III pre daily stimulation scores from 7-month MDS-UPDRS III pre daily stimulation scores. The descriptive results indicate minimal changes.

5 Supplementary Videos

Supplementary Video 1 Caption

Video 1 displays vCR treatment effects in 3 patients from study 1. The 1st patient's baseline walking recording is shown off medication and then exercising on medication after 6 weeks of vCR treatment. The 2nd patient is shown walking off medication at baseline and after 3 months of vCR therapy. The 2nd patient is also shown doing exercises on medication after 5 months of vCR therapy and after 6 months of vCR therapy and a pre-planned 1-month vCR pause in stimulation on medication. In the 3rd patient, we show acute vCR effects in the patient's walking and postural stability examined during the 1st visit of treatment off medication (baseline vs after 4 hours of vCR treatment).

Supplementary Video 2 Caption

Video 2 displays vCR treatment effects in patient 2 from baseline to 6 days of vCR therapy. Patient 2 is on medication in all recordings.

References

Bi, G. Q., & Poo, M. M. (1998). Synaptic modifications in cultured hippocampal neurons: dependence on spike timing, synaptic strength, and postsynaptic cell type. *Journal of Neuroscience*, *18*(24), 10464-10472.

Burkitt, A. N., Meffin, & Grayden, D. B. (2004). Spike-Timing-Dependent Plasticity: The Relationship to Rate-Based Learning Models with Weight Dynamics Determined by a Stable Fixed Point. *Neural Computation*, *16*(5), 885-940.

Cholewiak, R. W., & Collins, A. A. (1991). "Sensory and physiological bases of touch", in The psychology of touch, eds. M. A. Heller & W. Schiff (Hillsdale, NJ: Lawrence Erlbaum Associates, Inc.), 23-60.

Edwards, C., & Marks, R. (1995). Evaluation of biomechanical properties of human skin. *Clinics in Dermatology*, *13*(4), 375-380.

Franke, E. K. (1951). Mechanical impedance of the surface of the human body. *Journal of Applied Physiology*, *3*(10), 582-590.

Jones, L. A., & Lederman, S. J. (2006). *Human hand function*. New York, NY: Oxford University Press.

Kromer, J. A., Khaledi-Nasab, A., & Tass, P. A. (2020). Impact of number of stimulation sites on long-lasting desynchronization effects of coordinated reset stimulation. *Chaos: An Interdisciplinary Journal of Nonlinear Science*, *30*(8), 083134.

Kromer, J. A., & Tass, P. A. (2020). Long-lasting desynchronization by decoupling stimulation. *Physical Review Research*, *2*(3), 033101.

Lederman, S. J., & Klatzky, R. L. (2009). Haptic perception: A tutorial. *Attention, Perception, & Psychophysics*, *71*(7), 1439-1459.

Manos, T., Zeitler, M., & Tass, P. A. (2018). How stimulation frequency and intensity impact on the long-lasting effects of coordinated reset stimulation. *PLoS Computational Biology*, *14*(5), e1006113.

Markram, H., Lübke, J., Frotscher, M., & Sakmann, B. (1997). Regulation of synaptic efficacy by coincidence of postsynaptic APs and EPSPs. *Science*, *275*(5297), 213-215.

Moore, T. J., & Mundie, J. R. (1972). Measurement of specific mechanical impedance of the skin: Effects of static force, site of stimulation, area of probe, and presence of a surround. *The Journal of the Acoustical Society of America*, *52*(2B), 577-584.

Mortimer, B. J., Zets, G. A., & Cholewiak, R. W. (2007). Vibrotactile transduction and transducers. *The Journal of the Acoustical Society of America*, *121*(5), 2970-2977.

Ocker, G. K., Litwin-Kumar, A., & Doiron, B. (2015). Self-organization of microcircuits in networks of spiking neurons with plastic synapses. *PLoS Computational Biology*, *11*(8), e1004458.

Phillips, J. R., & Johnson, K. O. (1981). Tactile spatial resolution. III. A continuum mechanics model of skin predicting mechanoreceptor responses to bars, edges, and gratings. *Journal of Neurophysiology*, *46*(6), 1204-1225.

Popovych, O. V., & Tass, P. A. (2012). Desynchronizing electrical and sensory coordinated reset neuromodulation. *Frontiers in Human Neuroscience*, *6*, 58.

Song, S., Miller, K. D., & Abbott, L. F. (2000). Competitive Hebbian learning through spike-timing-dependent synaptic plasticity. *Nature Neuroscience*, *3*(9), 919-926.

Vallbo, A. B., & Johansson, R. S. (1984). Properties of cutaneous mechanoreceptors in the human hand related to touch sensation. *Human Neurobiol*ogy, *3*(1), 3-14.
